# Metagenomic analysis of the gut microbiome composition associated with vitamin D supplementation in Taiwanese infants

**DOI:** 10.1038/s41598-021-82584-8

**Published:** 2021-02-03

**Authors:** Wei-Te Lei, Kai-Yao Huang, Jhih-Hua Jhong, Chia-Hung Chen, Shun-Long Weng

**Affiliations:** 1 Department of Pediatrics, Hsinchu Mackay Memorial Hospital, Hsinchu City, 300 Taiwan; 2 Department of Medicine, Mackay Medical College, New Taipei City, 252 Taiwan; 3 Department of Medical Research, Hsinchu Mackay Memorial Hospital, Hsinchu City, 300 Taiwan; 4 Department of Computer Science and Engineering, Yuan Ze University, Taoyuan City, 320 Taiwan; 5 Department of Obstetrics and Gynecology, Hsinchu Mackay Memorial Hospital, Hsinchu City, 300 Taiwan; 6 Mackay Junior College of Medicine, Medicine, Nursing and Management College, Taipei City, 112 Taiwan

**Keywords:** Microbial communities, Classification and taxonomy, High-throughput screening

## Abstract

Early childhood is a critical stage for the foundation and development of the gut microbiome, large amounts of essential nutrients are required such as vitamin D. Vitamin D plays an important role in regulating calcium homeostasis, and deficiency can impair bone mineralization. In addition, most people know that breastfeeding is advocated to be the best thing for a newborn; however, exclusively breastfeeding infants are not easily able to absorb an adequate amount of vitamin D from breast milk. Understanding the effects of vitamin D supplementation on gut microbiome can improve the knowledge of infant health and development. A total of 62 fecal sample from healthy infants were collected in Taiwan. Of the 62 infants, 31 were exclusively breastfed infants and 31 were mixed- or formula-fed infants. For each feeding type, one subgroup of infants received 400 IU of vitamin D per day, and the remaining infants received a placebo. In total, there are 15 breastfed and 20 formula-fed infants with additional vitamin D supplementation, and 16 breastfed and 11 formula-fed infants belong to control group, respectively. We performed a comparative metagenomic analysis to investigate the distribution and diversity of infant gut microbiota among different types of feeding regimes with and without vitamin D supplementation. Our results reveal that the characteristics of infant gut microbiota not only depend on the feeding types but also on nutrients intake, and demonstrated that the vitamin D plays an important role in modulating the infant gut microbiota, especially increase the proportion of probiotics in breast-fed infants.

## Introduction

Vitamin D has aroused great interest based on the recognition that vitamin D deficiency can impair bone mineralization, especially in high-risk groups, including infants, children, pregnant women and lactating mothers, or elderly^1–5^. The most well-known function of vitamin D is to regulate the balance of calcium and phosphate to maintain human bone health. Otherwise, vitamin D plays an important role in both the immune and cardiovascular systems, it is considered to be a kind of steroid hormone^6^. Several studies have revealed the high prevalence of poor vitamin D status in school-age children around the world^7–12^, and few studies investigated the nutritional vitamin D status of newborns^13,14^. The main reasons for low level of vitamin D in children are without supplementary feeding and lack of outdoor sun exposure^15^. However, rickets and osteomalacia are exactly caused by insufficient vitamin supplementation^16–19^. Besides, it can lead to a lack of calcium absorption and affect phosphorus metabolism^20^, which increase the risk of cancer development, including multiple myeloma, prostate cancer and ovarian cancer^21^. It also associated with immune system diseases, metabolic syndrome^22^, and even complications of pregnancy^19^. The island of Taiwan lies across the Northern Tropic, which has more than 1,400 h of sunshine per year; however, these two evidences have shown that a substantial proportion of children in Taiwan have suboptimal levels of vitamin D, and its relationship with various allergic diseases^23,24^. Evidence has accumulated regarding the effects of vitamin D supplements during pregnancy on postpartum fetal vitamin D levels^25,26^, and other studies have described the association of vitamin D deficiency with many diseases in infants such as skeletal integrity, bone mass, asthma, type 1 diabetes and impaired lung function^27–30^. Early childhood is a critical stage for the foundation and development of both host and the gut microbiome, large amounts of essential nutrients are required, including certain vitamins, minerals, protein, fats, water, and carbohydrates. Dietary intake is one of the major determinants of health, it has been commonly assumed that breast milk is the ideal food for infants by providing the combination of essential nutrients; however, vitamin D in breast milk is often not enough to supply the nutrition need for infants^31,32^. The most widely accepted approach to get additional vitamin D is through supplementation^33^. In order to prevent the vitamin D deficiency in infants, the American Academy of Pediatrics (AAP) has updated the recommendations regarding vitamin D supplementation, a minimum daily intake of 400 IU of vitamin D is necessary for infants^34,35^.

The gastrointestinal microbiota plays a key role in human health, which participates in many important biochemical reactions such as vitamin and other nutrients biosynthesis, metabolism of drug and xenobiotics, regeneration of epithelial cells, storage of fats and mature of immune system^36^. Human gut microbiome can help its hosts extract dietary energy more efficiently by providing metabolic activities within the intestine^37,38^. Other recent studies have also suggested the diet and dietary supplements can influence the gut microbiota composition, thereby changing the activity of gut microbiota and linking with obesity traits^39–41^. In recent years, the relationship between human gut microbiome and health has been a topic of concern worldwide. The term “brain-gut-microbiome axis” is used to refer to the role of the dynamic changes in the gut microbiota can alter brain physiology and behavior^42–44^. There are increasing evidence showing that human gut microbiome not only influences gastrointestinal tract within the loop system, but also the other organs or tissues, such as the brain, lung, kidneys as well as in skin^45–48^. Human gut microbial ecology is dynamic in infancy, which has a significant impact on the development of the digestive system and host immune system, and also associated with the allergic diseases. Establishing the gut microbiota during infancy is a vital determinant of an individual's life-long health^49^. However, it is not clear yet whether the role of vitamin D in the infant gut microbiota. But a previous study found that vitamin D involved in modulation of innate immunity by regulating the antimicrobial peptides (AMP)^50^, and it has been demonstrated that the relative composition of the intestinal microbiota of mouse was influenced by the level of AMP expression^51^. Therefore, it suggests that the composition of the intestinal microbiota could be influenced by vitamin D status. To investigate the gut bacterial communities structure and diversity in host, there are many studies used the 16S rRNA high-throughput sequencing to analyze the microbial community in the gastrointestinal tract^52–56^, and even especially in the infant's gastrointestinal tract^57–62^.

Most people know that breastfeeding is advocated to be the best thing for a newborn; however, recent studies have shown a large proportion of pregnant and breastfeeding mothers having suboptimal vitamin D status, these women may not be able to support the breast milk with an adequate amount of vitamin D^63–66^. However, due to the relationships between vitamin D status and gut microbiome signatures in infants have not been satisfactorily examined. Therefore, the aim of this study was to determine the effect of vitamin D supplementation on gut microbiome development in Taiwanese infants. We performed a comparative metagenomic analysis of fecal samples to investigate the distribution and diversity of infant gut microbiota among different types of feeding regimes with and without vitamin D supplementation.

## Methods

### Ethics approval and consent to participate

This study was reviewed and approved by the Ethics Committee of MacKay Memorial Hospital, Taiwan (No: 17MMHIS088e). All of the methods were performed in accordance with relevant guidelines and regulations, including any relevant details. The written informed consent was provided by each parent of the infant, as approved by the Institutional Review Board.

### Study participants

A total of 62 healthy infants have been recruited into the population who were born at the Hsinchu Mackay memorial hospital in Taiwan, of which 29 are males and 33 are females (Table 1). All of infants were recruited from birth to 4 months, and informed consent was obtained from all guardians. Of the 62 infants, 31 were exclusively breastfed infants (BFI) and 31 were mixed- or formula-fed infants (FFI). For each feeding type, one subgroup of infants received 400 IU of vitamin D per day, and the remaining infants received a placebo. In total, there are 15 breastfed (BFVD) and 20 formula-fed infants (FFVD) with additional vitamin D supplementation, and 16 breastfed (BFCT) and 11 formula-fed infants (FFCT) belong to control group, respectively, and the detailed information is shown Table S1. Infants were excluded if they had any other sources of nutrition, dietary restrictions (e.g. hypoallergenic formula), consumed higher density formula (greater than 20 cal/ounce), had exposure to antibiotics, or had any gastrointestinal infection or disease that affected the integrity of the intestinal mucosa.

**Table 1:** Characteristics of the study population, n = 62, given as median, interquartile ranges, or percentage.

Characteristics	Newborn	1-month-old	4-month-old
Sex (M/F)		29 / 33	
VD investigation (ng/mL)	16.6 (8.5–36.5)	–	36.1 (4.1–109)
Birth weight (kg)	3.03 (2.04–3.86)	4.2 (3.3–5.2)	6.8 (5.3–8.4)
Birth length (cm)	50 (45–54)	54 (47–58.1)	63 (59.3–69)
Exclusively breastfed	31 (50.0%)	31 (50.0%)	27 (43.5%)
Mixed fed	26 (41.9%)	26 (41.9%)	22 (35.5%)
Exclusively formula-fed	5 (8.1%)	5 (8.1%)	13 (21.0%)

### Fecal sample collection

The fecal samples were obtained from healthy term infants delivered vaginally. Guardians gave written informed consent for fecal samples of infants, and clinical data were also collected such as demographic information, maternal and paternal age at infant’s birth, delivery method, height and weight of infant, and maternal over-the-counter or prescription medications taken during pregnancy. Subsequently, the fecal samples were collected by Sigma-Transwab (Medical Wire), and then were temporarily stored at -4 °C before DNA extraction.

### DNA extraction and 16S ribosomal DNA sequencing

With reference to our previous work^67^, QIAamp DNA Stool Mini Kit (Qiagen) was used for DNA extraction of stool samples. The DNA was eluted with Buffer AE and centrifuged, after which the DNA extract was stored at − 20 °C until further analysis. The PCR primers were designed to amplify the V4 region of the bacterial 16S ribosomal DNA as described by Caporaso et al.^68^. PCR amplification was performed in 2X Taq Master Mix (Thermo Scientific). Amplicons were purified using the AMPure XP beads (Agencourt) and quantified using the Qubit dsDNA HS Assay Kit (Thermo Fisher Scientific), all according to the respective manufacturers’ instructions. For V4 library preparation, Illumina adapters were attached to the amplicons using the Illumina TruSeq DNA Sample Preparation v2 Kit. Purified libraries were processed for cluster generation and sequencing using the MiSeq system.

### Quality control for 16S rDNA sequencing data

To assess the composition of microbial communities from clinical samples, the V3-V4 region of the bacterial 16S rRNA gene was amplified with barcoded primers and sequenced as above.

According to the specific barcodes at the 5′ end of the sequence, all of the sequencing reads were divided into different samples. Paired-end sequences were generated by Illumina sequencing in FASTQ format, and the corresponding paired-end reads can be merged into a fragment. The version 1.9.1 of the Quantitative Insights Into Microbial Ecology (QIIME)^69^ pipeline (http://qiime.org/index.html) was applied for sequence quality assessment with the quality threshold is 19 (it meant Q20) and the ratio of mismatch is less than 10%. After filtering, the bases at the end of the sequence with lower quality (< 20) would be trimmed, and the length of sequence less than 100 nt were also excluded. Both forward- and reverse-sequencing reads after trimming that met the criteria were retained for analysis.

### Taxonomy assignment

Next, the filtered reads were taxonomically classified into OTU against the GreenGenes database (release 2013–08) for analysis and metabolic potential of the microbial community. Greengenes is a highly cited full-length 16S rRNA gene database in Bacteria and Archaea (https://greengenes.secondgenome.com/)^70^, since it is the default database in the pipeline. To taxonomically classify 16S rDNA fragments, the filtered reads were classified into OTU (Operational taxonomic unit) against the database with 97% phylogenetic similarity, and the sequences classified as unknown were removed from further analysis. After taxonomy assignment, an OTU table was generated that gives the number of reads per sample per OTU.

### Metagenome functional content prediction

To further study the biological function of the metagenomics, PICRUSt v1.1.4 (http://picrust.github.io/picrust/)^71^ was used to predict the functional composition of metagenomes bases on OTU table. Metabolic pathways analysis for the abundances of genes was performed with reference to the KEGG ortholog groups (KOs). OTU data generated in QIIME for all 16S rRNA datasets was used to prepare BIOM files formatted as input for PICRUSt and PICRUSt-predicted metagenomes based on OTUs marker gene sequences were estimated using default parameters. We furthered our study through detection of taxonomic clades, KEGG orthologs and KEGG modules that are significantly over/under-represented (or differentially abundant) in the infant environments through statistical analyses carried out on the inferred relative abundances.

### Identification of metagenomic biomarkers

To identify differentially abundant features that can be used as potential metagenomic biomarkers, the procedure of linear discriminant analysis (LDA) effect size was employed through LEfSe v1.0 (https://huttenhower.sph.harvard.edu/galaxy/)^72^ to identify the specific KOs/microbes that were differentially distributed between two conditions. For this analysis, the value of 0.05 was set for the alpha parameter significance threshold for the Krushkal-Wallis (KW) test and the log value of 1.0 was set for the LDA score cut-off^73^.

### Statistical analysis

The Shannon’s diversity index was calculated to determine the species richness as well as the evenness of distribution in the community, and the Chao1 index was calculated to estimate the total number of OTUs based on the actual observed species number. The differences between continuous variables in the different groups were contrasted by the non-parametric test of Mann–Whitney for two groups, and by the test of Kruskal–Wallis for more than two groups with a significant threshold of 0.05. In addition, one-way analysis of similarity (non-parametric test ANOSIM) was performed to test for statistically significant differences between the metagenomic profiles and *p*-values < 0.05 were considered to be significant.

### Availability of data and materials

The supporting data are available to researchers in the NCBI Sequence Read Archive, under study accession number PRJNA644496.

## Results

### Study population

As shown in Table 1, the physical and feeding characteristics are listed and the detailed information is reported in the Table S1, a total of 62 healthy individuals were enrolled, including 29 males and 33 females. Median serum concentration of 25-hydroxyvitamin D (25(OH)D) were 16.6 nanograms/milliliter (ng/mL) at the birth and 36.1 at 4-month-old of age. Nearly half of the mothers were exclusively breastfeeding at the first day after birth, the mixed and formula-fed infants were 26 (41.9%) and 5 (8.1%), respectively. At the age of 4-month-old, 4 exclusively breastfed infants switched to mixed feeding, and 8 from mixed feeding to exclusively formula feeding.

The serum concentration of 25(OH)D is a biological indicator that can be used to determine whether a patient is vitamin D deficient, while the normal concentration of vitamin D in newborn infants is above 20 ng/mL, and less than 12 ng/mL indicates high risk of vitamin D deficiency^74^. The results of the statistical analysis of serum 25(OH)D concentrations are given in Table 2, the mean level in BFCT, BFVD, FFCT and FFVD groups were 17.4, 19.3, 15.1 and 15.4 ng/mL at the birth, respectively; additionally, a total of 9 (14.5%) infants had vitamin D insufficiency and deficiency at the birth (Table S1). At the 4-month-old visit, the results of the vitamin D tests showed that, except in the BFCT group, the infants tend to have higher serum levels (above 20 ng/mL) compared to birth levels. From this, the evidence suggests that the supply of vitamin D from breast milk is limited. However, note that the mean value from 19.3 up to 59.6 in the BFVD group during the period, and up to 50.8 ng/mL in the FFVD group. The results indicated that there was a statistically significant difference (*p*-value < 0.0001) in serum concentration of 25(OH)D between the infants with and without vitamin D supplementation.

**Table 2:** Mean serum 25(OH)D levels of the infants with and without vitamin D supplementation.

Exposure	No. infants	25(OH)D Level at NB, mean ± SD (ng/mL)	p-value†	25(OH)D Level at 4 M, mean ± SD (ng/mL)	p-value†
BFCT	16	17.4 ± 5.5	0.1510	15.5 ± 8.7	< 0.0001
BFVD	15	19.3 ± 7.7		59.6 ± 18.4	
FFCT	11	15.1 ± 5.7		25.7 ± 3.5	
FFVD	20	15.4 ± 6.4		50.8 ± 14.0	

*25(OH)D: 25-hydroxyvitamin D; ^†^Kruskal–Wallis test was used to measure variance in mode of diet.

### Comprehensive characterization of bacterial community composition in infants

To characterize the infant gut microbiome with and without vitamin D supplements, 16S rRNA gene sequencing of was used to investigate 62 fecal samples from healthy infants after a normal term pregnancy. The fecal samples were collected longitudinally at birth (NB), 1 month (1 M) and 4 month (4 M) of age, and analyzed for the bacterial community constitutions. In total, 24,119,684 paired-end sequencing reads of high-quality sequences were generated (average 129,675 reads per sample). After quality filtering, resulted in 20,163,971 (average 83.1%) sequencing reads which used for further analysis. 18,343,693 reads (average 75.6%) were aligned to 16S rRNA gene sequences with a similarity higher than 97%, these sequences were assigned to 7,432 OTUs and clustered into 378 genera of 24 phyla.

**Figure 1:**
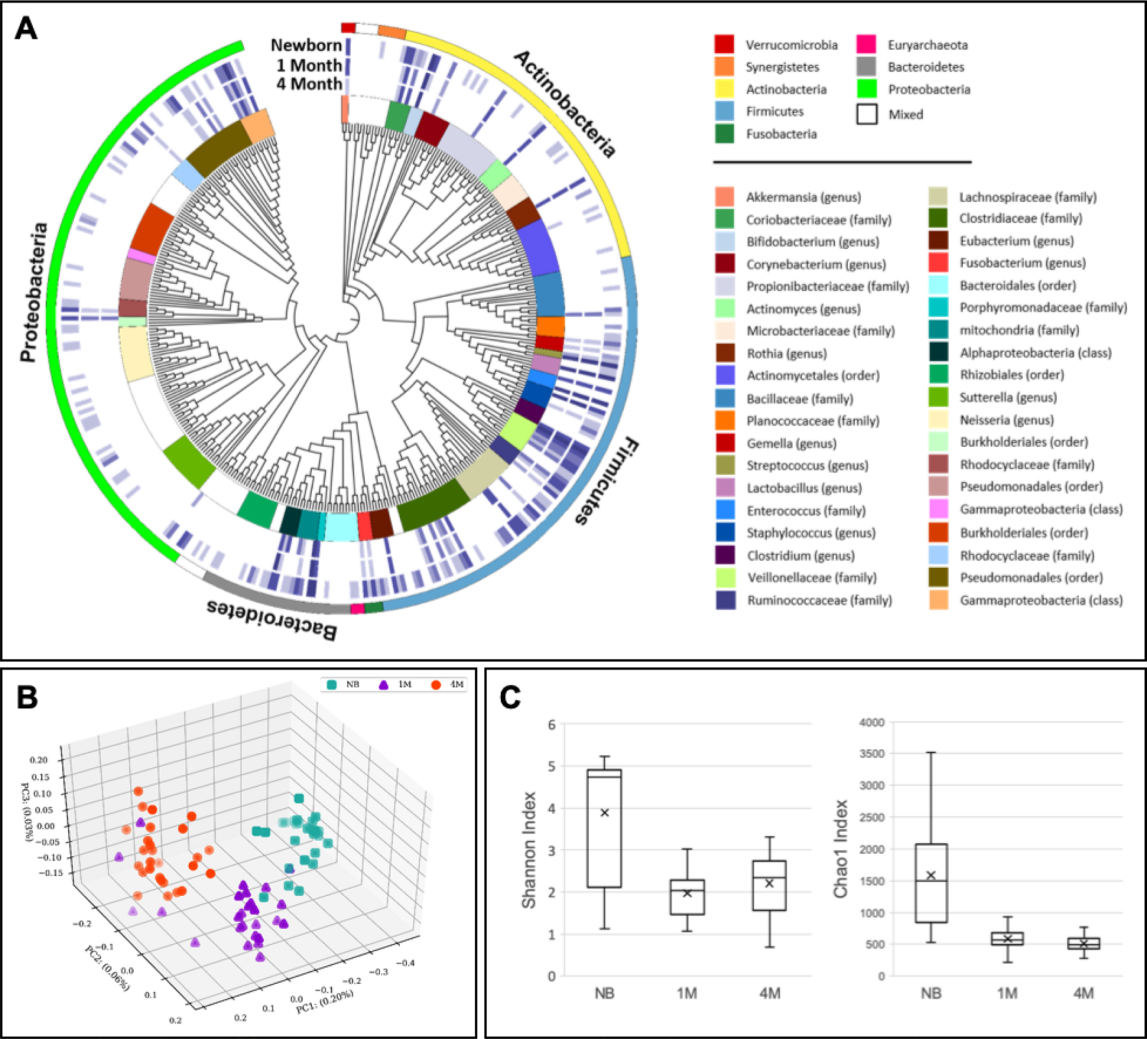
Differences in the fecal microbial communities of newborns, 1 M and 4 M infants. (**A**) Representative commensal gut microbial community in infants during first 4 months. The colored blocks outermost indicate phyla and the heatmap circles show absolute abundances of each OTUs in the newborns, 1-month-old and 4-month-old infants. The innermost neighbor-joining phylogenetic tree containing one representative of each of the OTUs. (**B**) PCoA analysis plot based on unweighted UniFrac distances, each point represents the microbiota of a single sample, and colors reflect metadata for that sample. (**C**) Comparison of bacterial diversity across sample groups in Shannon’s diversity index and Chao1 index.

As shown in Fig. 1A (also see Table S2), the phylogenetic tree of representative sequences from dominant OTUs which was created by Circos v0.64 (http://circos.ca/)^75^, indicating that the community structure of the gut microbiota in Taiwanese infants aged between 0, 1 and 4 months. *Firmicutes* and *Actinobacteria* were the most prevalent phyla, followed by *Bacteroidetes* and *Proteobacteria*; these results were consistent with previous studies that infant gut microbiota is dominated by these bacteria^60,76,77^. Notable, the phylum of *Proteobacteria* presented a higher proportion at the birth compared to other time points. Similar trend was observed by previous study that the family *Enterobacteriaceae* of the phylum *Proteobacteria* was the dominant bacteria of the infant gut microbiota in the first week of newborn life, which then decreases over time with a concomitant increase of *Bifidobacterium*^78^.

To better reveal the differences of the microbial communities among the different time points, Principal Coordinate Analysis (PCoA) of unweighted UniFrac^79^ distances was performed to assess the beta diversity of communities. The result showed that the gut bacteria composition differed significantly at the different time points is shown in the Fig. 1B (ANOSIM, *R* = 0.742, *p* = 2.14E-05). The microbiota composition in the NB samples was significantly different from that of the 1 M and 4 M groups, while the 1 M and 4 M samples were more similar to each other, consistent with the observation in Fig. 1A and the previous research studies^76,80^.

Additionally, biodiversity analysis was performed for comparing the richness and evenness of infant gut microbial communities between the different time points. We found that the alpha diversity index strongly reduced in infants during the period from birth to term age, while have no obvious change after 1 month (Fig. 1C, Table 3, and also see Table S3). These findings suggest that the infant gut microbiota structure is instability and dynamic at birth, and it is widely assumed that the early establishment of the infant gut microbiome has been associated with numerous factors including the modes of delivery, the types of infant feeding, and probiotic and prebiotic use^81^.

**Table 3:** Richness and diversity of fecal microbiota in infants, by early-life exposures.

Exposure	No. infants	Diversity index*, mean ± SD	p-value†	Richness score*, mean ± SD	p-value†
Overall	62	2.7 ± 1.3		886.1 ± 636.1	
**Sex**					
Male	29	2.4 ± 1.2	0.7091	614.4 ± 645.4	0.6029
Female	33	2.4 ± 1.2		623.4 ± 646.8	
**Feeding type**					
Breastfeeding (BF)	31	2.3 ± 1.2	0.907	573 ± 629.1	0.1358
Formula feeding (FF)	31	2.5 ± 1.2		654.3 ± 980.5	
**Diet at NB**					
BFCT	16	4.7 ± 1.4	0.5263	1551 ± 642	0.9696
BFVD	15	4.8 ± 1.2		1688.1 ± 727.6	
FFCT	11	4.7 ± 1.2		1295.6 ± 980.5	
FFVD	20	4.6 ± 1.2		1266.8 ± 694.6	
**Diet at 4 M**					
BFCT	16	1.9 ± 0.4	0.0325	478.6 ± 116.2	0.4853
BFVD	15	2.3 ± 0.6		463.8 ± 118.2	
FFCT	11	2.8 ± 1.3		607.7 ± 684	
FFVD	20	2.3 ± 0.6		501.9 ± 130.5	

*Diversity was measured by Shannon index, which evaluates both the number of species and evenness of each group. Richness was measured by Chao1 score, which evaluates the number of different species present; ^**†**^Mann–Whitney two-tailed test was used to measure variance in sex, antibiotics treatment group, which Kruskal–Wallis test was used to measure variance in mode of diet.

### Identification of the differences in gut microbial communities between breast- and formula-fed Infants

To better identify the effects of different infant feeding regimes on the initial establishment of gut microbiota, we analyzed the impact between breast and formula feeding in vaginally delivered infants. Of the 27 fecal samples from infants under different types of feeding without any other nutrients, 16 breastfed infants (BFI) and 11 formula-fed infants (FFI) were available for the evaluation of microbial communities.

**Figure 2:**
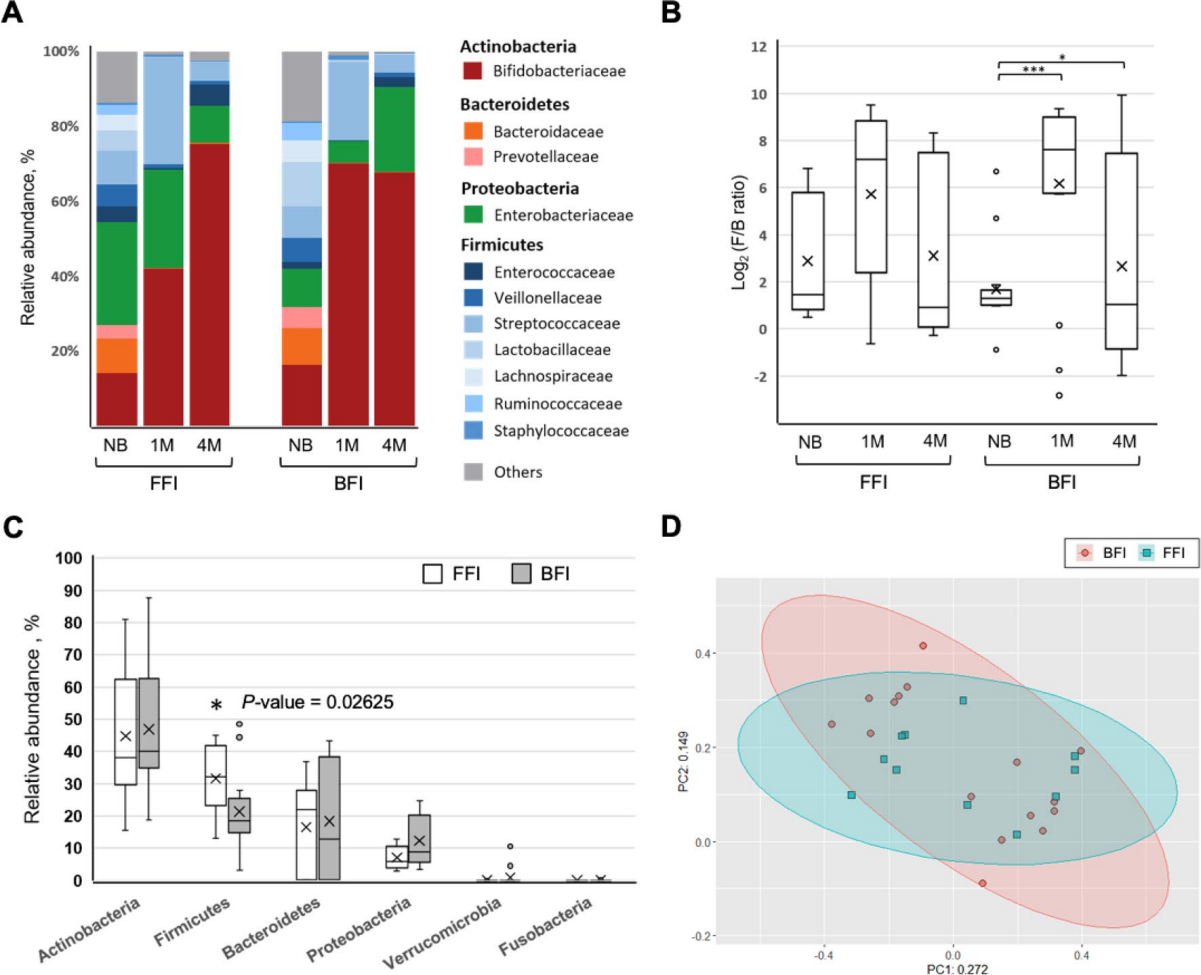
Comparison of relative abundances of microbial taxa between breast- and formula-fed infants. (**A**) Taxonomic distribution of bacterial communities at the family level (**B**) Box plot of log F/B ratio of the BFI and FFI groups. (**C**) Relative abundances of bacterial phyla within fecal microbiota of BF and FF infants at 4-month-old of age. (**D**) Principal Co-ordinate Analysis based on unweighted UniFrac distance of the OTUs in BFI and FFI samples at 4-month-old of age. Pairwise comparisons using Mann–Whitney test. *P < 0.05. **P < 0.0005. ***P < 0.00001.

As shown in Fig. 2A, the results revealed 3 main bacterial phyla in guts microbiota in both feeding groups during the period from birth to 4 months of age, including *Actinobacteria*, *Firmicutes* and *Proteobacteria*. However, no matter BFIs or FFIs, the family *Bifidobacteriaceae* belong to the phylum *Actinobacteria* which have the highest relative abundance at 1 month and 4 months of age. In fact, several studies have indicated that a stable gut microbiota is established after two big transitions in infancy^82–84^; the first transition results in *Bifidobacteriaceae*-dominant microbiota soon after birth, and the second transition results in the establishment of an adult-type complex microbiome dominated by the phyla *Bacteroidetes* and *Firmicutes* during the weaning period until three years of age. Interestingly, the results showed that the first transition of the gut microbiota in BFIs were earlier than in FFIs.

Besides, the log-transformed ratio of *Firmicutes* to *Bacteroidetes* (F/B) ratio in feces was measured for different types of feeding, which shows that the ratio of BFI group was obviously higher than FFI group at 1 month, and then greatly decrease at 4 months. Some studies have demonstrated that the higher F/B ratio in the gut microbiota is associated with obesity and many diseases in human^85,86^. A previous study has suggested that breastfed infants gain weight more rapidly than the formula-fed peers during the first 2–3 months of life and then taper off^87^, which is consistent with our observation shown in Fig. 2B.

To explore the difference in dominant bacterial community compositions between two feeding groups before solids, Fig. 2C presents the relative abundance of OTUs at the phylum level in 4-month-old infants. Here, we found that a high level of phylum *Actinobacteria* was observed in both groups, but there is no statistically significant difference between the two groups. Some strains of *Bifidobacteriaceae* family are considered as important probiotics^88^. The *Bifidobacteria*-dominated gut community provides colonization resistance to pathogens^89^, enhances immune surveillance^90^, reduces inflammation^91^, and improves mucosal gut barrier function^92^. We also observed that the phylum *Firmicutes* showed a significant difference (*p*-value = 0.02625) between the two groups, FFIs have a significantly higher level of Firmicutes. Otherwise, a few studies have conducted that the family *Enterobacteriaceae* belong to the phylum *Proteobacteria* were found to a slightly higher relative abundance in breastfed infants^93–96^. *Enterobacteriaceae* are opportunistic pathogens but rarely produce human disease; however, it could be a health risk to infants who use the contaminated products^97^. Principal Co-ordinate Analysis based on unweighted UniFrac distance of the OTUs at 4-month-old of age showed that the samples clustered according to BFI and FFI groups, and indicated that no significant differences existed between the two feeding types (Fig. 2D).

### The impact of vitamin D supplements on breastfed infant gut microbiota

It has been known that a low vitamin D concentration in human breast milk which causes bone weakness and a higher risk of fracture in infants at birth. Due to the effects of vitamin D deficiency in breastfed infant gut microbiota are still unknown. Here, we compared the gut microbial composition in the fecal samples of breastfed infants with and without vitamin D supplementation at the 4 months of age (BFVD and BFCT group).

**Figure 3:**
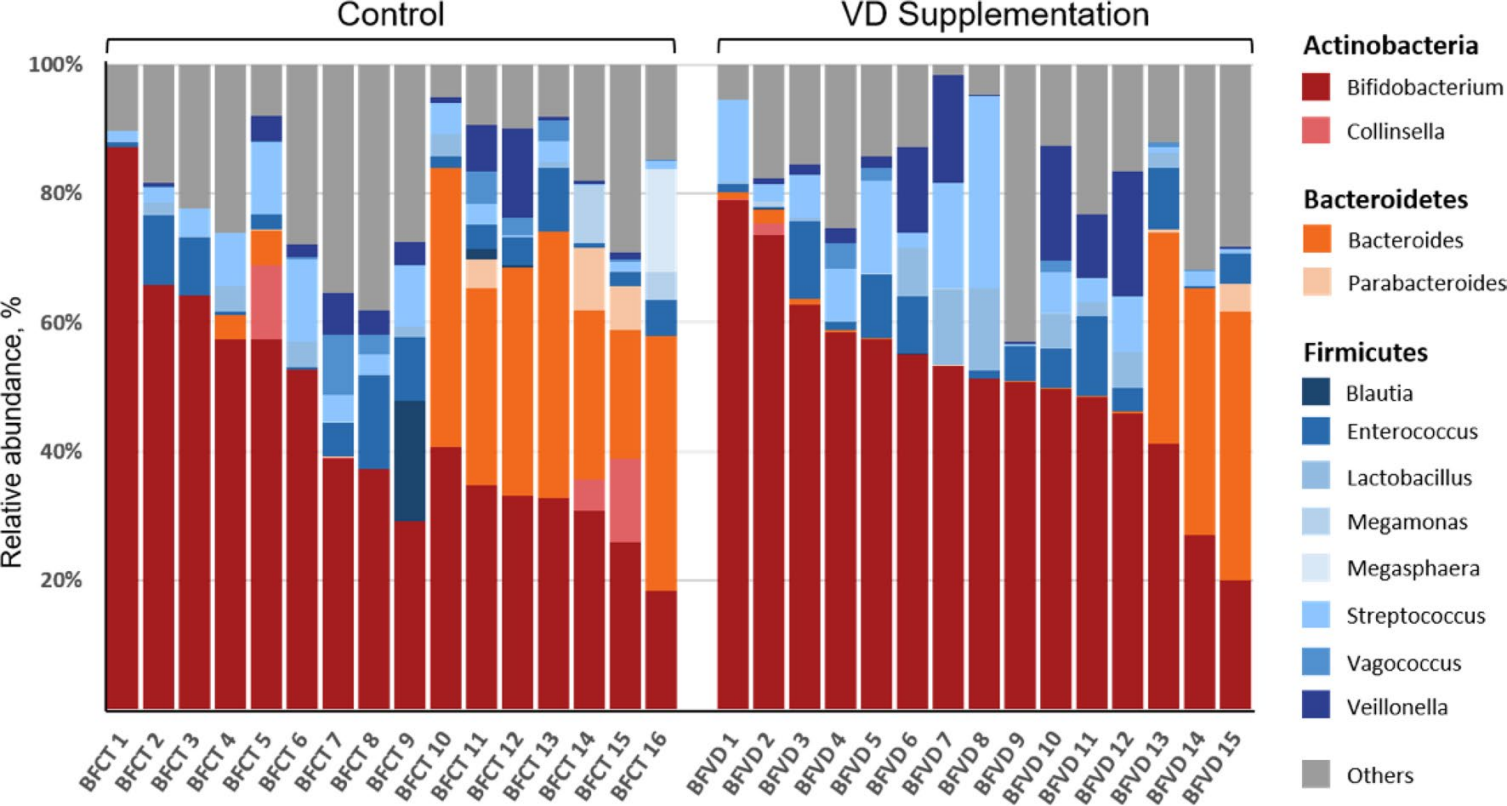
Taxonomic distribution of bacterial communities in fecal samples from breastfed infants with vitamin D supplementation.

Figure 3 (also see Table S4) show the bacterial taxonomy assignments at the genus level of two groups, we observed that *Bifidobacterium* was the dominant genus across all samples of two groups, and the proportion accounts for more than half of the total intestinal bacteria. It is worth noting that a significant correlation between the proportion of *Bifidobacterium* and BFVD group, almost all the samples in the group contain a higher proportion of *Bifidobacterium* than non-VD group, the proportion of *Bifidobacterium* in BFVD and BFCT were 51.6% and 44.2% of average, respectively. *Bifidobacterium* is one of the most common probiotics in the intestinal tract of mammals, which is a lactic acid bacteria (LAB)^98,99^. The population as the dominant group in infant fecal microbiota and as a conserved feature during early gastrointestinal tract colonization that have been demonstrated in previous studies^100^. Additionally, the genus *Streptococcus* (7.7% vs. 4.5%) and *Lactobacillus* (3.3% vs. 1.0%) also have higher abundance in BFVD versus BFCT group. In contrast, the genus *Bacteroides* was observed with lower abundances in the BFVD group instead of pure breastfed infants without vitamin D supplementation (7.9% vs. 15.4%).

**Figure 4:**
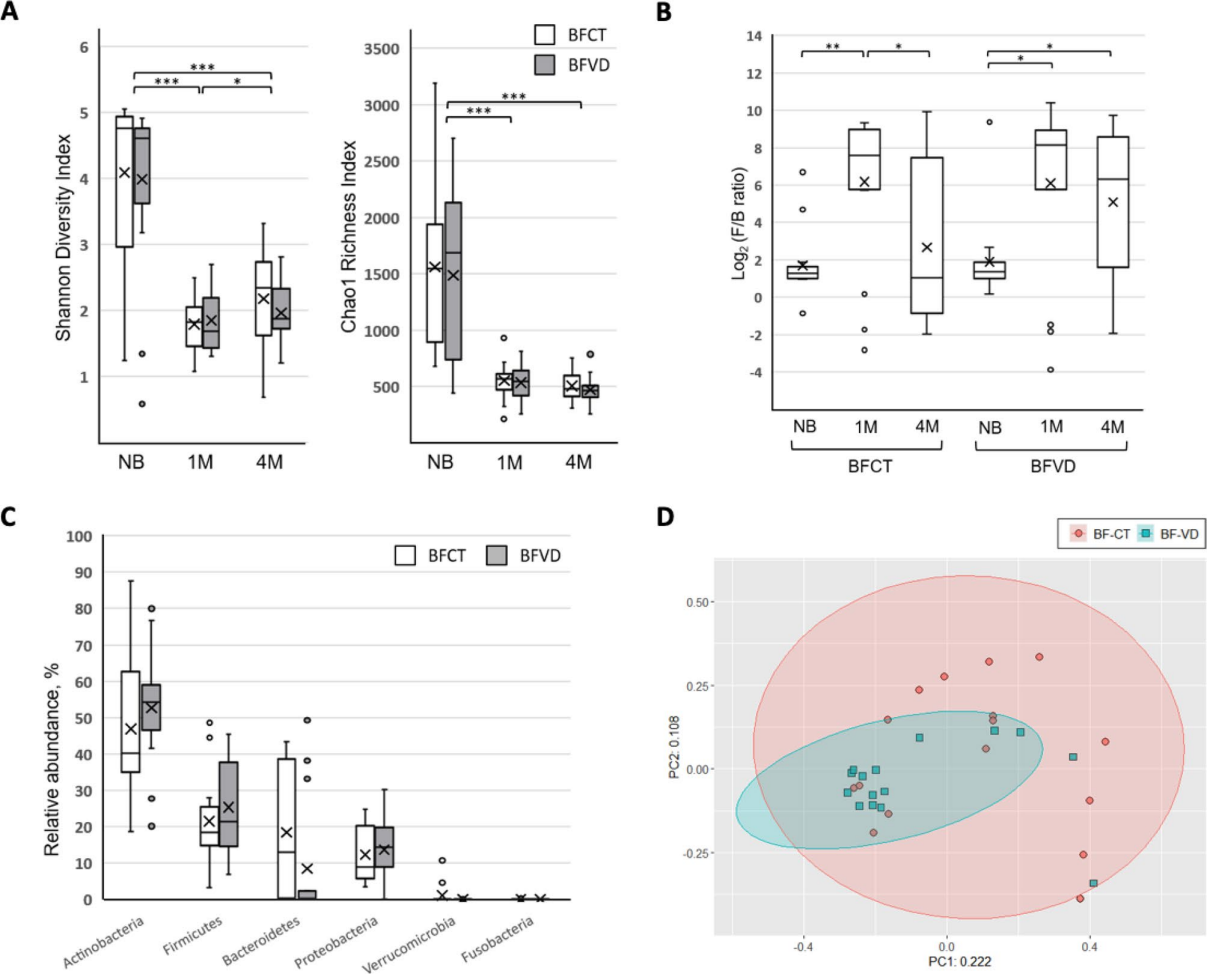
Comparison of relative abundances of microbial taxa between breastfed infants with and without vitamin D supplementation. (**A**) Diversity and richness of bacterial communities in the BFCT and BFVD groups. (**B**) Box plot of log F/B ratio of the BFCT and BFVD groups. (**C**) Relative abundances of bacterial phyla within fecal microbiota of BFCT and BFVD infants at 4-month-old of age. (**D**) Principal Co-ordinate Analysis based on unweighted UniFrac distance of the OTUs in BFCT and BFVD samples at 4-mont h-old of age. Pairwise comparisons using Mann–Whitney test. *P < 0.05. **P < 0.0005. ***P < 0.00001.

The Shannon’s diversity indexes for the BFVD and BFCT samples on each time point (during the period from birth to 4 M of age) are shown in Fig. 4A (also see Table S3). The highest indices were shown in both of the two groups at the birth, and subsequently decreased dramatically at the 1-month-old of age. When we compared BFCT to BFVD in terms of the indexes at the 4-month-old, the former had a higher value than latter. It is worth highlighting that the BFVD group had a higher F/B ratio than BFCT group especially at the 4-month-old of age (Fig. 4B, also see Table S6). In general, the F/B ratio is considered a potential biomarker of pathological conditions in human health^101,102^, and some articles indicate that the ratio is directly related to the BMI centile^103^. The median ratio of BFCT group significantly decreased at the 4 months is closely linked to the previous finding, the breastfed infants only grow more rapidly in first 2–3 months of age and less rapidly after 3 months^104^.

For further analysis, the relative OTU abundances of major bacteria phyla at 4-month-old were calculated and shown in Fig. 4C. However, except *Actinobacteria* and *Bacteroidetes*, there were no significant differences in relative abundance of *Firmicutes*, *Bacteroidetes*, *Proteobacteria*, *Verrucomicrobia*, and *Fusobacteria* between two groups. Figure 4D shows the PCoA analysis of unweighted UniFrac distances was used to calculate pairwise distances between the bacterial communities of BFVD and BFCT groups (R = 0.089, *p* = 0.047), it seems that the infants' vitamin D status was positively associated with the gut microbial community.

### Metabolic characterization and functional biomarkers in the fecal samples from breastfed infants with vitamin D supplementation

**Figure 5:**
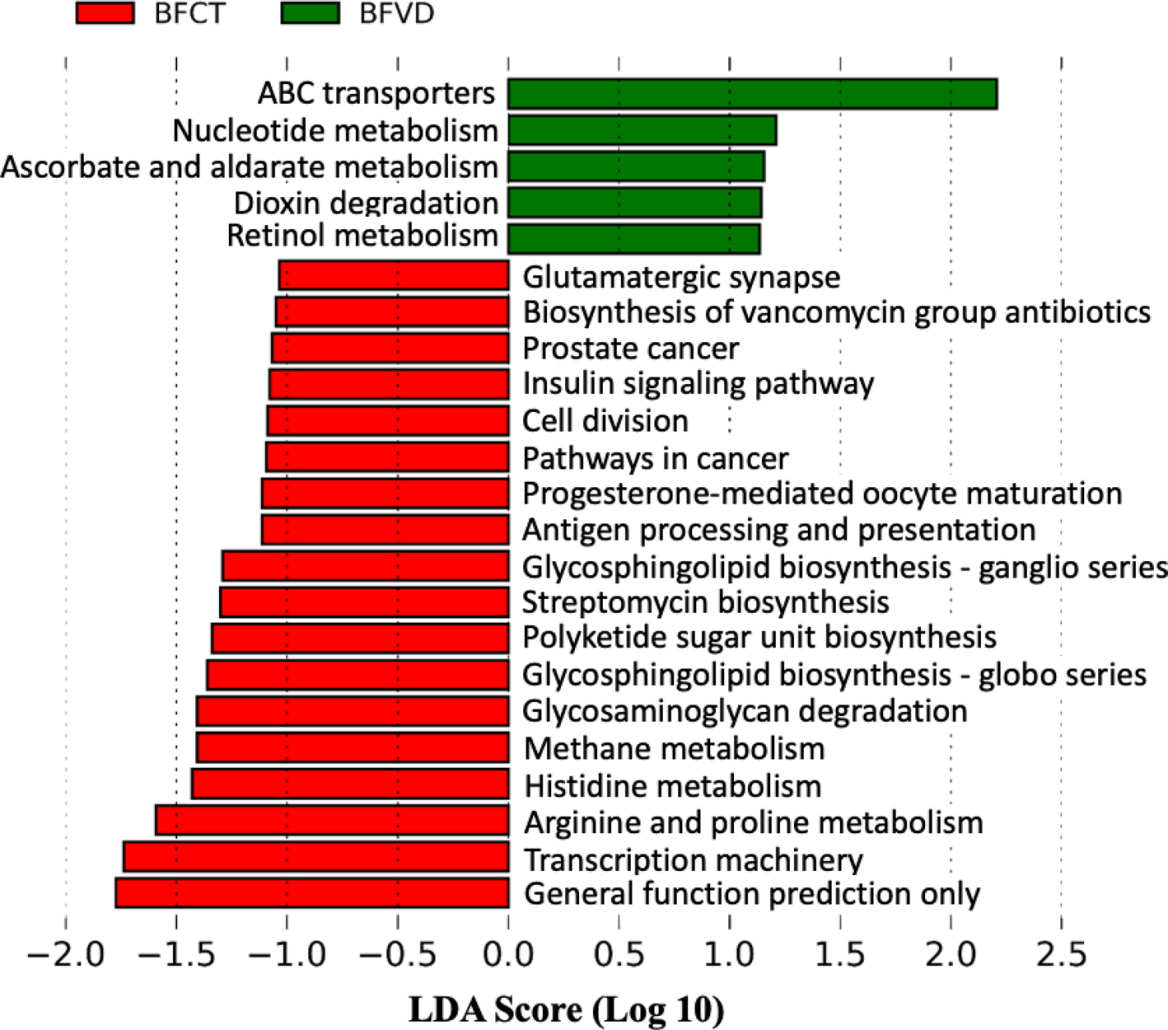
Inference of metagenomic functional content of gut microbiota from breastfed infants with vitamin D supplementation.

To understand the metabolic potential of vitamin D deficiency and identifying differentially abundant functional features, the PICRUSt tool was used to predict the metagenome functional content of BFVD and BFCT groups. Predicting metagenomic function based on the KEGG database by PICRUSt, a total of 23 KEGG metabolism pathway were predicted across all samples of the two compared groups (Fig. 5, also see Table S7).

Most of the pathways identified in the BFVD group are necessary for sustenance of life, including ABC transporters, Nucleotide metabolism, Ascorbate and aldarate metabolism, Dioxin degradation. It is worth noting that, the results show that enriched levels of Retinol metabolism in BFVD groups, with implications in several essential developmental processes such as vision, bone, and teeth (Table S7). On the contrary, in the case of the BFCT group, most of the pathways identified were found to be involved in various kinds of biosynthesis and antibiotic producing modules, such as Glycosphingolipid biosynthesis, Polyketide sugar unit biosynthesis, Biosynthesis of vancomycin group antibiotics, and Streptomycin biosynthesis. Finally, numerous modules describing metabolic processes were identified to be over-represented in both two groups. All of these modules are essential in affect microbial distribution, survival, and proliferation of microbes in the environment.

**Figure 6:**
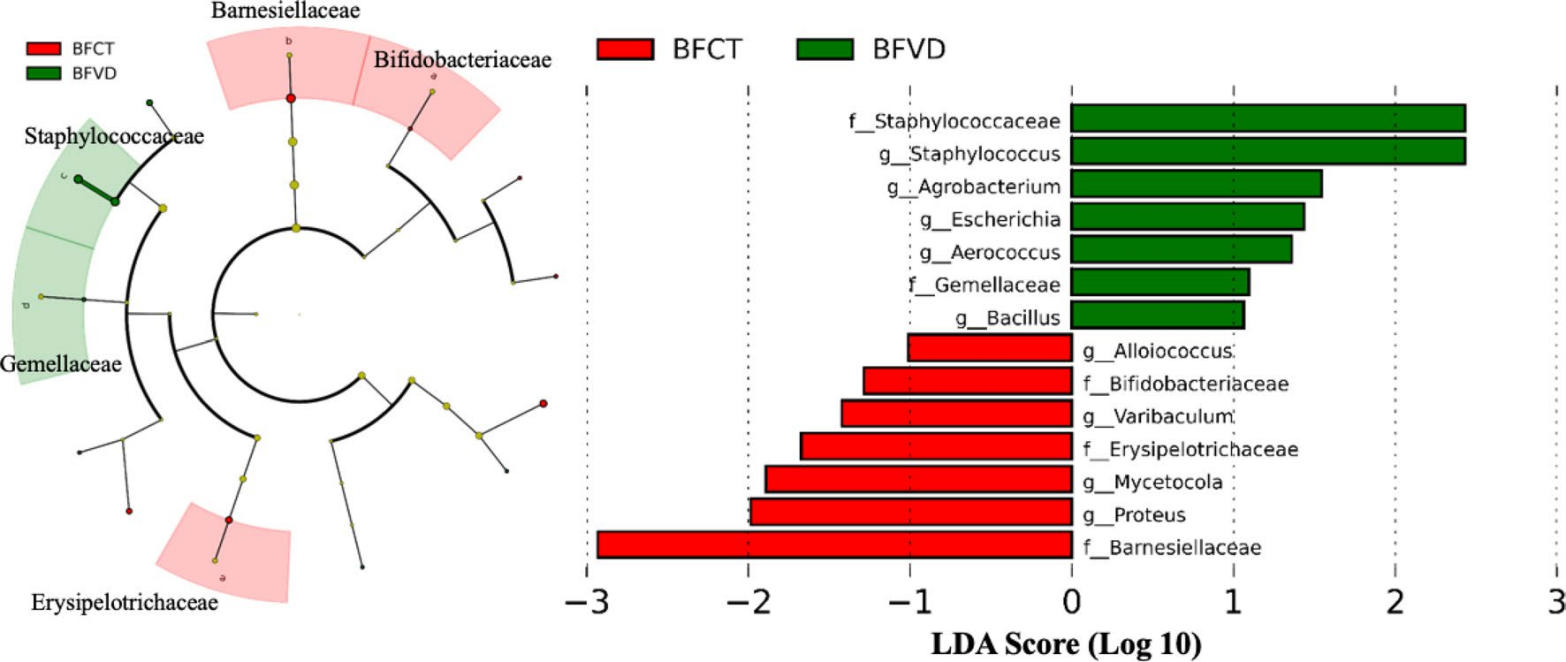
Identification of the most differentially taxa between breastfed infants with vitamin D supplementation.

In addition, we also compared the fecal microbiota in BFCT and BFVD groups using LEfSe to identify the specific bacterial taxa associated with vitamin D intake. The greatest differences in multiple levels of taxa between the two communities were shown in taxonomic cladogram (Fig. 6). The result indicated the significantly decreased phylum *Firmicutes* and its genus can be one of the biomarkers of BFVD group, including the genus *Staphylococcus*, *Aerococcus* and *Bacillus*; likewise, the genus *Agrobacterium* and *Escherichia* of the phylum *Proteobacteria* can be the biomarker. In contrast, family *Bifidobacteriaceae* and *Erysipelotrichaceae* can be the biomarkers of BFCT group.

However, in BFCT group, due to a lack of some essential nutrients in breast milk such as vitamin D and iron, these bacteria probably will not have a significant growth advantage in the environment already undergoing natural selection. Ultimately, we observed that the gut microbiota normally exists in a stable state when vitamin D sufficient, most of genes are known to involve in catabolism and anabolism pathways.

### Detections of associations of vitamin D supplementation in formula-fed infants

In order to further ensure no matter what kind of feeding, whether the gut microbiota of formula-fed infants can be affected through sufficient vitamin D intake. Hence, we compared the gut microbiota composition in formula-fed infants with and without sufficient vitamin D (FFVD).

**Figure 7:**
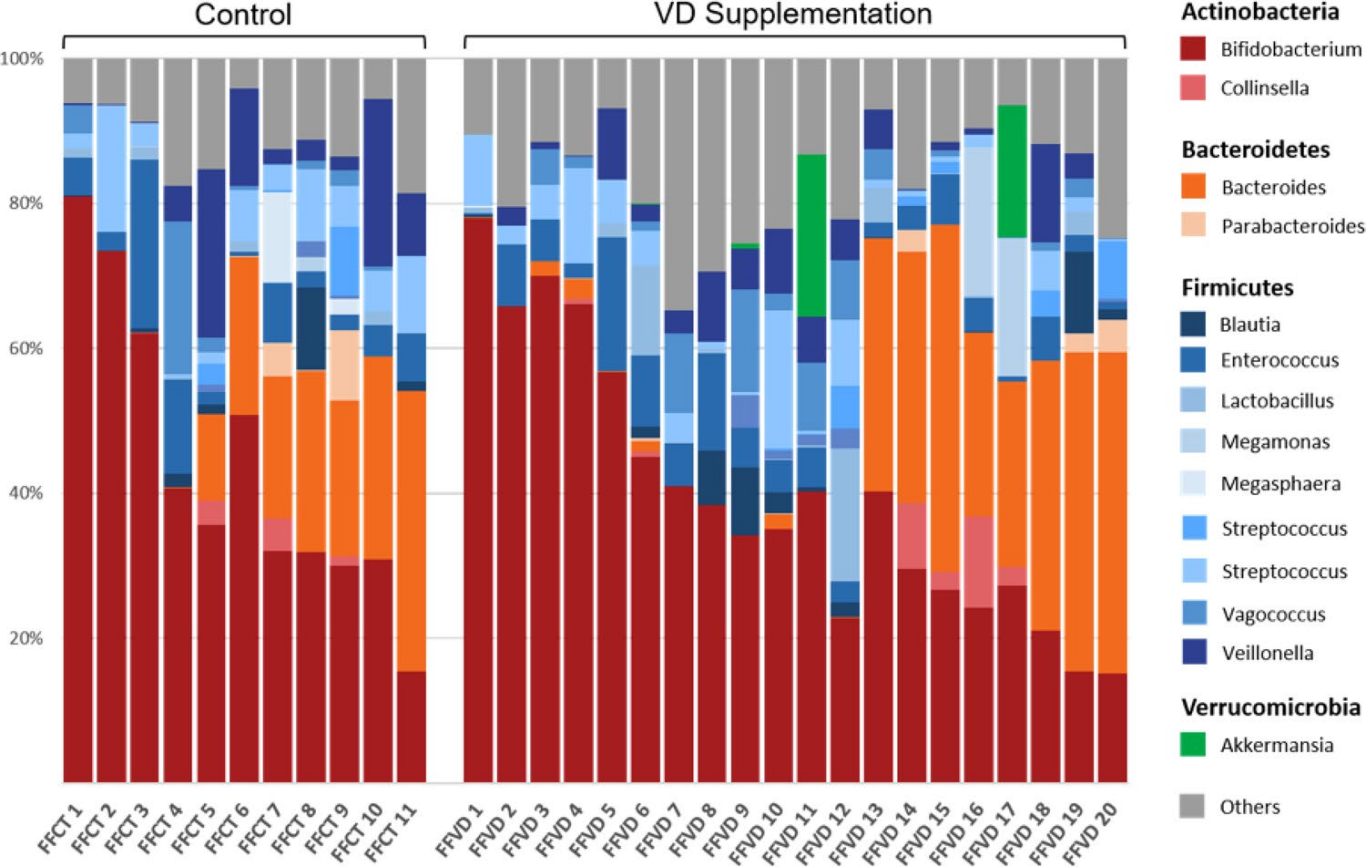
Taxonomic distribution of bacterial communities in fecal samples from formula-fed infants with vitamin D supplementation.

Figure 7 shows the similar microbiota at the genus level, which consisted mainly of the phylum *Actinobacteria* and its genus *Bifidobacterium*, and the genus *Bacteroides* were detected a higher proportion in part of samples from both two groups. Unfortunately, there is no significant difference of gut microbiota profile between FFCT and FFVD.

**Figure 8:**
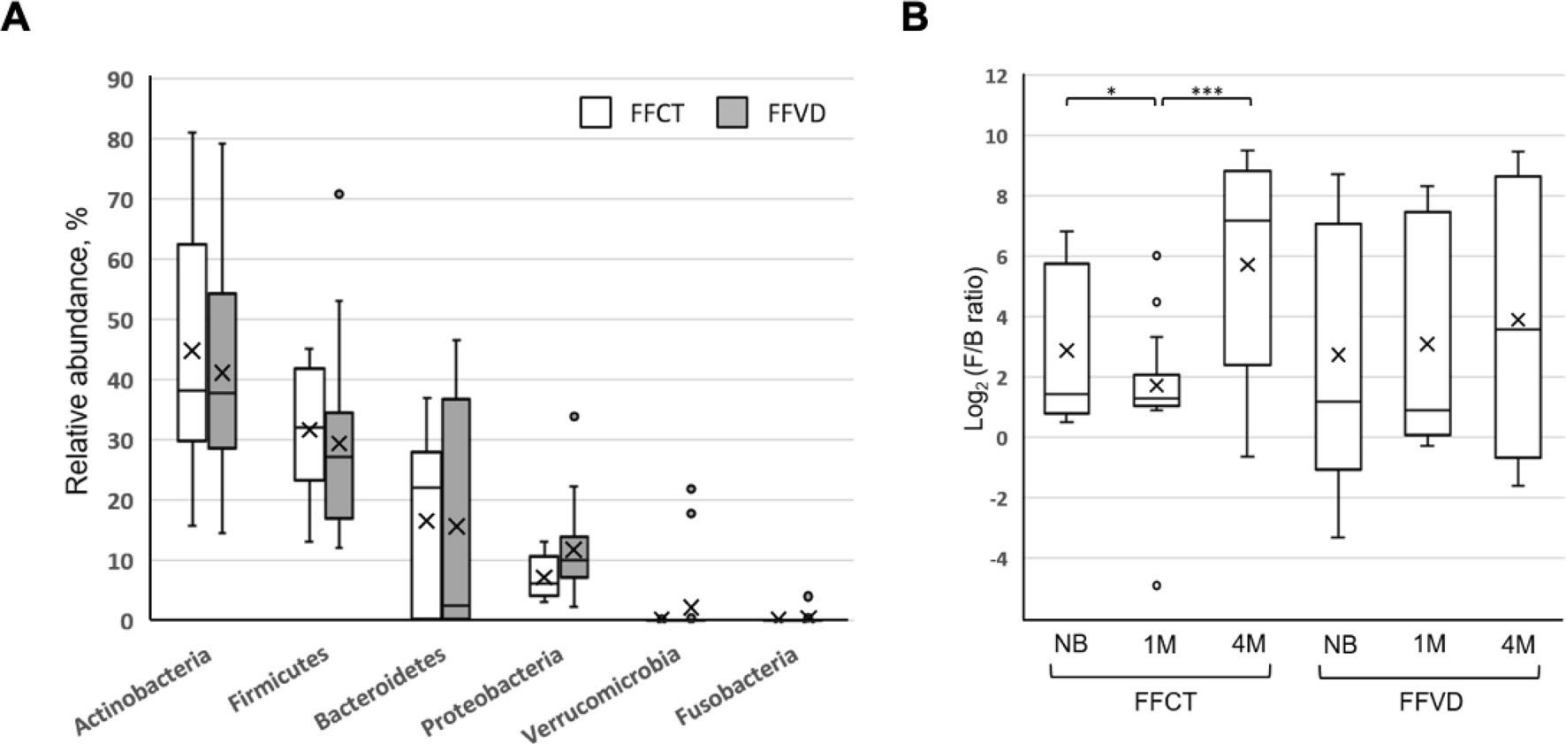
Comparison of relative abundances of microbial taxa between formula-fed infants with and without vitamin D supplementation. (**A**) Relative abundances of bacterial phyla within fecal microbiota of FFCT and FFVD infants at 4-month-old of age. (**B**) Box plot of log F/B ratio of the FFCT and FFVD groups. Pairwise comparisons using Mann–Whitney test. *P < 0.05. **P < 0.0005. ***P < 0.00001.

As shown in Fig. 8A,B, based on previous observation in breastfed infant with sufficient vitamin D, while no similar effects on gut microbiota composition and activity were observed in the FFVD group. However, this is probably due to the formula milk contains a variety of nutrients like fat, phosphorus, sodium, potassium, iron, calcium, zinc and multivitamin which is necessary for the growth of the child, as driving factors of gut microbiota changes and have impact on health.

## Discussion

The objective of this study was to investigate that the influence of vitamin D supplementation on gut microbiota in breast- and formula-fed infants during the early months of a newborn’s life. Although many experts advocated that breast milk is the best food for infants, but a lack of some essential nutrients (in particular vitamin D and iron) that can affect growth and development of the child adversely, and even affect the gut microbiota composition and function. Thus, we provide comprehensive metagenomic profiles of fecal samples to investigate the distribution and diversity of infant gut microbiota among different types of feeding regimes with and without vitamin D supplementation.

Metagenomic analysis results showed that the phylum *Proteobacteria* presented a higher proportion at the birth. Subsequently, bacteria from *Actinobacteria* were the most abundant phylum present in both breast- and formula-fed infants, which then decreases over time. Early than formula-fed infants, the proportion of family *Actinobacteria* in the breastfed infants was significantly increased at 1-month-old of age, of particular interest is the presence of the *Bifidobacteria*, considered probiotic microorganisms useful to the host for their beneficial effects. It is worth noting that, no matter breastfeeding or formula feeding, with a sufficient level of vitamin D, the gut microbiota normally exists in a stable state which are very similar to each other.

**Figure 9:**
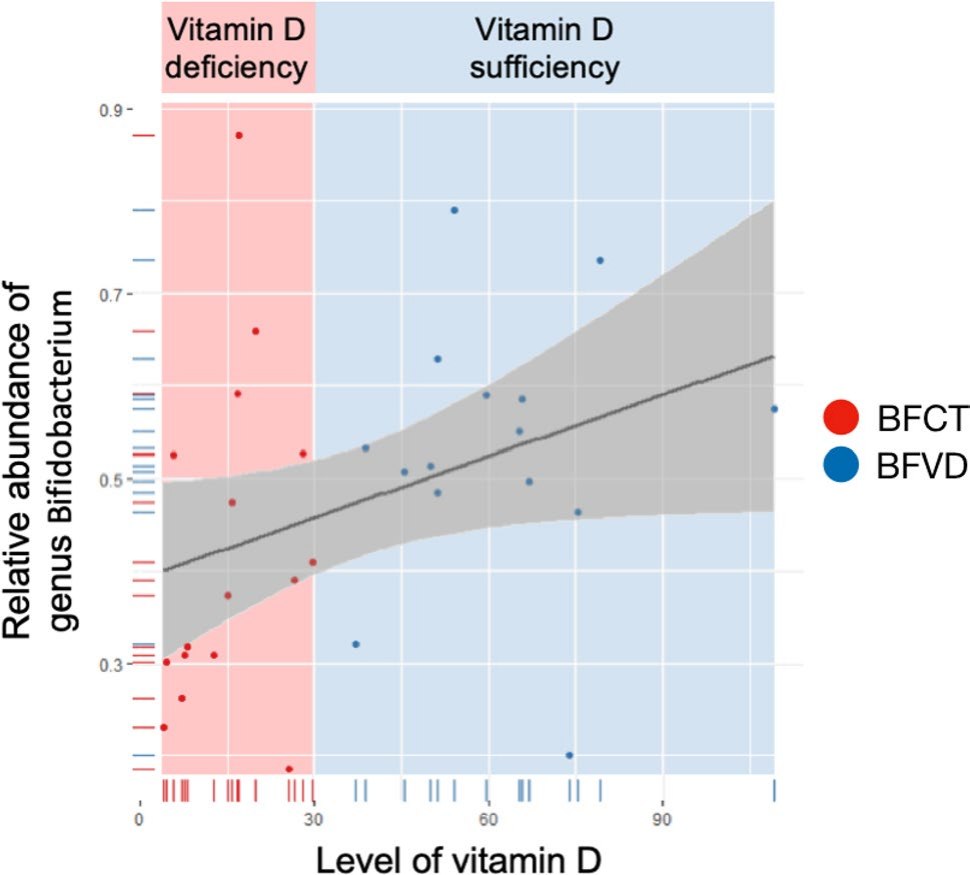
Linear regression results of abundance of *Bifidobacterium* and circulating vitamin D level.

**Figure 10:**
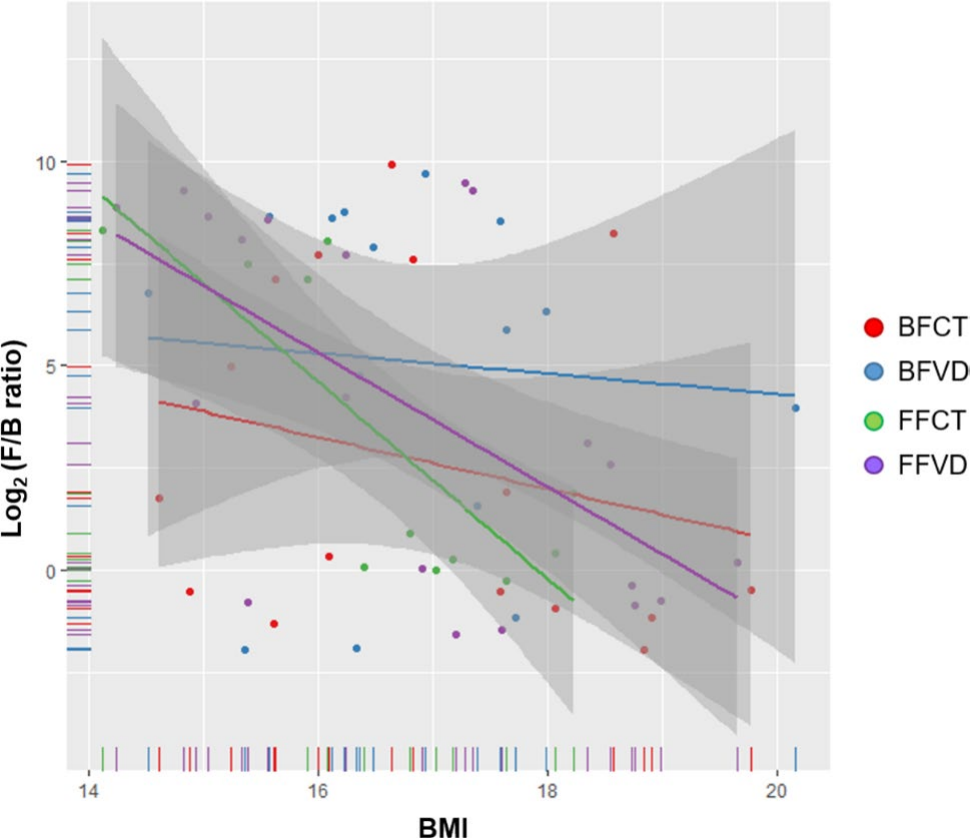
Linear regression results between BMI and F/B ratio at 4-month-old of age in 4 groups of diet.

Moreover, as shown in Fig. 9, the proportion of *Bifidobacterium* was positively correlated with that of circulating vitamin D level in breastfed infants. *Bifidobacterium*, the probiotics can secrete the antimicrobial compounds and metabolites to fight against the various diseases and gastrointestinal disorders. Meanwhile, the F/B ratio was significantly negatively correlated with BMI at 4-month-old of age, especially in FFI groups as shown in Fig. 10 (FFCT: *p*-value = 0.003805, FFVD: *p*-value = 0.00428), and the phenomenon was possibly prevented by vitamin D supplementation. Furthermore, the metabolic analysis profiles in breastfed infant with vitamin D deficiency reveal that microbial communities live in highly competitive surroundings, because most of the pathways identified were found to be involved in various kinds of antibiotic biosynthesis. However, since the formula milk contains a variety of nutrients, we could not observe the similar effects above mentioned in formula-fed infants with vitamin D supplementation.

Our results reveal that the characteristics of infant gut microbiota not only depend on the feeding types but also on nutrients intake, and demonstrated that the vitamin D plays an important role in modulating the human gut microbiota, especially increase the proportion of probiotics in infants.

## Supplementary Information

Supplementary Information.
