# Supplementary material for: Metagenomic analysis of the gut microbiome composition associated with vitamin D supplementation in Taiwanese infants
Source: Sci Rep. 2021 Feb 3;11:2856. doi: 10.1038/s41598-021-82584-8 (PMC7859236; doi:10.1038/s41598-021-82584-8)
Supplement: Supplementary file 1 — Supplementary Information. [file 41598_2021_82584_MOESM1_ESM.docx]

**Metagenomic Analysis of the Gut Microbiome Composition Associated with Vitamin D Supplementation in Taiwanese Infants**

**Wei-Te Lei**^1,+^**, Kai-Yao Huang**^2,3,+^**, Jhih-Hua Jhong**^4^**, Chia-Hung Chen**^3^**, and Shun-Long Weng**^2,5,6,^*

^1^Department of Pediatrics, Hsinchu Mackay Memorial Hospital, Hsinchu City 300, Taiwan

^2^Department of Medicine, Mackay Medical College, New Taipei City 252, Taiwan

^3^Department of Medical Research, Hsinchu Mackay Memorial Hospital, Hsinchu City 300, Taiwan

^4^Department of Computer Science and Engineering, Yuan Ze University, Taoyuan City 320, Taiwan

^5^Department of Obstetrics and Gynecology, Hsinchu Mackay Memorial Hospital, Hsinchu City 300, Taiwan

^6^Mackay Junior College of Medicine, Medicine, Nursing and Management College, Taipei City 112, Taiwan

^*^Corresponding authors: [4467@mmh.org.tw](mailto:4467@mmh.org.tw) (Shun-Long Weng)

^+^these authors contributed equally to this work

**Supplementary Tables**

**Table S1: Descriptive Data of the Study Population**

| **ID** | **Class** | **Gender** | **Birth** | | | **4M** | | | **Vitamin D investigation** | |
| --- | --- | --- | --- | --- | --- | --- | --- | --- | --- | --- |
|  |  |  | **Weight (kg)** | **Height (cm)** | **BMI** | **Weight (kg)** | **Height (cm)** | **BMI** | **0M (ng/mL)** | **4M (ng/mL)** |
| VD08 | BFCT | girl | 2.492 | 49 | 10.38 | 6.5 | 60 | 18.06 | 12.4 | 15.2 |
| VD16 | BFCT | girl | 2.83 | 46 | 13.37 | 5.9 | 63 | 14.87 | 12.1 | 26.6 |
| VD20 | BFCT | boy | 2.882 | 47 | 13.05 | 5.8 | 63 | 14.61 | 27.5 | 7.6 |
| VD28 | BFCT | girl | 3.51 | 50 | 14.04 | 6.5 | 62.5 | 16.64 | 13.2 | 28 |
| VD29 | BFCT | girl | 2.906 | 48.5 | 12.35 | 7.2 | 61.7 | 18.91 | 19 | 5.8 |
| VD32 | BFCT | girl | 3.03 | 48 | 13.15 | 7.2 | 64 | 17.58 | 15.8 | 16.7 |
| VD36 | BFCT | boy | 3.376 | 50 | 13.50 | 6 | 62 | 15.61 | 13.9 | 19.8 |
| VD48 | BFCT | boy | 3.062 | 50 | 12.25 | 6.8 | 65 | 16.09 | 18.5 | 4.1 |
| VD49 | BFCT | girl | 2.774 | 49 | 11.55 | 7 | 63 | 17.64 | 26.2 | 4.7 |
| VD52 | BFCT | boy | 3.15 | 54 | 10.80 | 7.4 | 68 | 16.00 | 26.1 | 25.6 |
| VD55 | BFCT | boy | 3.208 | 51 | 12.33 | 6.3 | 64.3 | 15.24 | 10.4 | 29.7 |
| VD65 | BFCT | boy | 3.288 | 52 | 12.16 | 6.6 | 65 | 15.62 | 20.6 | 7.3 |
| VD83 | BFCT | boy | 3.094 | 50 | 12.38 | 8.1 | 64 | 19.78 | 16.3 | 17.1 |
| VD87 | BFCT | boy | 3.07 | 49 | 12.79 | 7 | 61.4 | 18.57 | 14.5 | 8.2 |
| VD89 | BFCT | girl | 3.204 | 53 | 11.41 | 7 | 64.5 | 16.83 | 18.9 | 12.7 |
| VD92 | BFCT | girl | 2.822 | 48 | 12.25 | 7.5 | 63.1 | 18.84 | 24.5 | 15.8 |
| VD26 | BFVD | girl | 3.422 | 51.5 | 12.90 | 5.9 | 60.1 | 16.33 | 17.1 | 109 |
| VD27 | BFVD | boy | 2.044 | 48 | 8.87 | 6.6 | 64 | 16.11 | 14.9 | 75.4 |
| VD38 | BFVD | girl | 3.178 | 50 | 12.71 | 7 | 63 | 17.64 | 16.6 | 79.2 |
| VD40 | BFVD | girl | 3.022 | 51 | 11.62 | 6.3 | 61 | 16.93 | 26.6 | 51.3 |
| VD41 | BFVD | boy | 2.86 | 51 | 11.00 | 6.7 | 64 | 16.36 | 23.4 | 51.1 |
| VD43 | BFVD | girl | 2.956 | 50 | 11.82 | 6.5 | 60.8 | 17.58 | 14.2 | 74 |
| VD46 | BFVD | girl | 3.86 | 52 | 14.28 | 7.5 | 61 | 20.16 | 22.4 | 59.6 |
| VD53 | BFVD | boy | 2.27 | 45 | 11.21 | 5.4 | 61 | 14.51 | 26.8 | 67.1 |
| VD66 | BFVD | boy | 2.786 | 47 | 12.61 | 6.2 | 63.1 | 15.57 | 36.5 | 65.3 |
| VD71 | BFVD | girl | 2.412 | 46 | 11.40 | 7 | 62.4 | 17.98 | 19.3 | 54.1 |
| VD73 | BFVD | boy | 3.428 | 52 | 12.68 | 7.4 | 67 | 16.48 | 28.4 | 65.9 |
| VD77 | BFVD | girl | 2.47 | 48 | 10.72 | 5.4 | 59.3 | 15.36 | 11 | 38.8 |
| VD80 | BFVD | boy | 3.25 | 49 | 13.54 | 6.9 | 62.4 | 17.72 | 15.6 | 50 |
| VD88 | BFVD | girl | 3.278 | 50 | 13.11 | 7.3 | 64.8 | 17.38 | 13.9 | 37.2 |
| VD91 | BFVD | boy | 2.908 | 50 | 11.63 | 6.5 | 63.3 | 16.22 | 33.5 | 45.5 |
| VD06 | FFCT | girl | 3.216 | 50 | 12.86 | 6.8 | 65.4 | 15.90 | 9.9 | 20.4 |
| VD11 | FFCT | boy | 2.968 | 49 | 12.36 | 6.4 | 64.5 | 15.38 | 15.1 | 20.2 |
| VD13 | FFCT | boy | 3.658 | 52 | 13.53 | 8.4 | 69 | 17.64 | 17.8 | 29 |
| VD14 | FFCT | girl | 2.938 | 49 | 12.24 | 5.9 | 60.6 | 16.07 | 11.2 | 21.8 |
| VD22 | FFCT | girl | 3.316 | 50 | 13.26 | 6.3 | 62 | 16.39 | 8.5 | 25.1 |
| VD33 | FFCT | boy | 3.264 | 50 | 13.06 | 7.1 | 64.3 | 17.17 | 15.3 | 28.7 |
| VD42 | FFCT | boy | 3.02 | 48 | 13.11 | 7.4 | 64 | 18.07 | 13.1 | 26.2 |
| VD44 | FFCT | girl | 3.298 | 51 | 12.68 | 7.3 | 63.3 | 18.22 | 11.6 | 25.7 |
| VD54 | FFCT | boy | 2.978 | 48 | 12.93 | 6.8 | 63.2 | 17.02 | 21.4 | 24.2 |
| VD57 | FFCT | boy | 3.062 | 50 | 12.25 | 7.1 | 65 | 16.80 | 25.1 | 29.2 |
| VD90 | FFCT | girl | 2.892 | 47 | 13.09 | 5.6 | 63 | 14.11 | 24 | 29.5 |
| VD02 | FFVD | girl | 3.258 | 50 | 13.03 | 6.4 | 61 | 17.20 | 11.2 | 41.6 |
| VD03 | FFVD | girl | 2.708 | 48 | 11.75 | 5.8 | 62.1 | 15.04 | 10.7 | 54.8 |
| VD07 | FFVD | boy | 2.81 | 52 | 10.39 | 6.7 | 67 | 14.93 | 13.6 | 56 |
| VD30 | FFVD | girl | 2.918 | 49 | 12.15 | 7.5 | 63.6 | 18.54 | 36.3 | 30.3 |
| VD31 | FFVD | boy | 2.726 | 49 | 11.35 | 7.9 | 67 | 17.60 | 26.6 | 70.4 |
| VD34 | FFVD | girl | 3.106 | 49 | 12.94 | 5.3 | 61 | 14.24 | 14.7 | 80.2 |
| VD47 | FFVD | girl | 3.046 | 50 | 12.18 | 7.2 | 62 | 18.73 | 13.6 | 41.3 |
| VD61 | FFVD | boy | 2.742 | 47 | 12.41 | 6.1 | 61.3 | 16.23 | 10.5 | 36.1 |
| VD62 | FFVD | girl | 2.652 | 47 | 12.01 | 6.9 | 60.3 | 18.98 | 17.1 | 49.8 |
| VD63 | FFVD | girl | 2.726 | 48 | 11.83 | 6.3 | 64 | 15.38 | 14 | 48.7 |
| VD64 | FFVD | boy | 3.778 | 52 | 13.97 | 7.1 | 64.1 | 17.28 | 17.4 | 59.4 |
| VD67 | FFVD | boy | 2.948 | 50 | 11.79 | 6.2 | 63.6 | 15.33 | 18.4 | 36.4 |
| VD72 | FFVD | girl | 3.306 | 52 | 12.23 | 7.8 | 65.2 | 18.35 | 18.8 | 67 |
| VD74 | FFVD | boy | 2.97 | 50 | 11.88 | 6.8 | 62.6 | 17.35 | 14.9 | 47 |
| VD76 | FFVD | girl | 2.664 | 50 | 10.66 | 6 | 60.8 | 16.23 | 27.5 | 30.2 |
| VD82 | FFVD | girl | 2.516 | 47 | 11.39 | 5.6 | 60 | 15.56 | 13.2 | 72.3 |
| VD85 | FFVD | boy | 3.214 | 49.5 | 13.12 | 7.8 | 63 | 19.65 | 16.8 | 45.5 |
| VD86 | FFVD | girl | 3.564 | 53 | 12.69 | 6.3 | 65.2 | 14.82 | 15.8 | 59.5 |
| VD93 | FFVD | girl | 2.94 | 49 | 12.24 | 6.5 | 62 | 16.91 | 13.3 | 60.4 |
| VD94 | FFVD | boy | 3.33 | 51 | 12.80 | 7.4 | 62.8 | 18.76 | 21.7 | 51.7 |

**Table S2: Major common bacteria in newborns, 1M and 4M infants.**

| **Genus** | **NB** | **1M** | **4M** |  | **Genus** | **NB** | **1M** | **4M** |
| --- | --- | --- | --- | --- | --- | --- | --- | --- |
| Collinsella | 26042 | 32416 | 22049 |  | SMB53 | 5037 | 1449 | 8772 |
| Eggerthella | 862 | 2281 | 6008 |  | Peptoniphilus | 917 | 1022 | 1056 |
| Bifidobacterium | 280076 | 1287836 | 1323992 |  | Finegoldia | 1467 | 2744 | 2409 |
| Corynebacterium | 3471 | 626 | 636 |  | Eubacterium | 20625 | 20497 | 29159 |
| Propionibacterium | 3834 | 5180 | 4561 |  | Fusobacterium | 19755 | 159 | 119 |
| Actinomyces | 12124 | 14608 | 9504 |  | Prevotella | 130847 | 9580 | 6616 |
| Rothia | 15768 | 31156 | 28096 |  | Bacteroides | 193348 | 197477 | 296570 |
| Bacillus | 254 | 1321 | 1143 |  | Parabacteroides | 12425 | 51909 | 15212 |
| Gemella | 3219 | 236 | 331 |  | Sutterella | 6056 | 3624 | 1307 |
| Lactococcus | 430 | 257 | 618 |  | Neisseria | 27061 | 189 | 137 |
| Streptococcus | 292995 | 599363 | 439064 |  | Haemophilus | 8579 | 165 | 251 |
| Lactobacillus | 245806 | 179238 | 121622 |  | Proteus | 132 | 2756 | 2859 |
| Vagococcus | 12552 | 14428 | 33922 |  | Serratia | 300 | 570 | 523 |
| Enterococcus | 130930 | 72439 | 106691 |  | Erwinia | 4624 | 1928 | 1889 |
| Staphylococcus | 22128 | 72996 | 65647 |  | Trabulsiella | 3483 | 456 | 459 |
| Clostridium | 5667 | 14100 | 11690 |  | Klebsiella | 130944 | 22778 | 22862 |
| Veillonella | 54787 | 113406 | 100161 |  | Enterobacter | 1654 | 736 | 820 |
| Megasphaera | 6128 | 18628 | 13807 |  | Akkermansia | 12512 | 23075 | 95 |
| Dialister | 4389 | 2325 | 759 |  | Porphyromonas | 12258 | 103 | 93 |
| Acidaminococcus | 1183 | 3362 | 1986 |  | Acinetobacter | 3139 | 116 | 94 |
| Phascolarctobacterium | 34987 | 382 | 220 |  | Atopobium | 12386 | 86 | 180 |
| Megamonas | 14197 | 2239 | 1556 |  | Granulicatella | 439 | 67 | 195 |
| Oscillospira | 5337 | 1225 | 3987 |  | Dorea | 5021 | 31 | 8359 |
| Faecalibacterium | 42812 | 240 | 174 |  | Anaerococcus | 708 | 98 | 124 |
| Ruminococcus | 25668 | 26700 | 16650 |  | Butyricimonas | 826 | 15 | 1792 |
| Blautia | 35721 | 252 | 1008 |  | Bilophila | 1724 | 19 | 582 |

**Table S3: The richness and diversity of microbial communities in each group.**

| **ID** | **Class** | **Shannon Index** | | | **Chao1 Index** | | |
| --- | --- | --- | --- | --- | --- | --- | --- |
|  |  | **0M** | **1M** | **4M** | **0M** | **1M** | **4M** |
| Breastfed infants without vitamin D supplementation, *n* = 16. | | | | | | | |
| VD08 | BFCT | 5.027248 | 1.304975 | 1.628973 | 1641.29 | 575.0189 | 754 |
| VD16 | BFCT | 1.121417 | 3.016993 | 2.354248 | 1492.966 | 569.5294 | 403.2826 |
| VD20 | BFCT | 1.924163 | 2.458027 | 1.553692 | 2374.853 | 514.0667 | 595.3061 |
| VD28 | BFCT | 4.828441 | 2.812154 | 1.794879 | 832.2118 | 623.5192 | 598.8077 |
| VD29 | BFCT | 4.830686 | 2.150541 | 1.198414 | 3189.563 | 464.2075 | 536.1765 |
| VD32 | BFCT | 2.107181 | 2.561408 | 1.544121 | 843.4167 | 591.1132 | 624.8837 |
| VD36 | BFCT | 3.43448 | 2.819978 | 1.923679 | 1588 | 559.8929 | 469.3 |
| VD48 | BFCT | 4.853337 | 2.042165 | 1.873866 | 1776.065 | 718.2031 | 498.575 |
| VD49 | BFCT | 4.535907 | 1.979172 | 1.718662 | 1734.414 | 569.6842 | 701.25 |
| VD52 | BFCT | 4.711135 | 2.05484 | 1.875052 | 1442.079 | 572.2174 | 398.9688 |
| VD55 | BFCT | 4.903527 | 1.978686 | 2.449031 | 2071.548 | 696.0154 | 313.5 |
| VD65 | BFCT | 4.618454 | 2.070837 | 1.94421 | 678.1207 | 464.6829 | 394.9268 |
| VD83 | BFCT | 4.855469 | 2.032978 | 1.42584 | 1513.925 | 212.1579 | 485.525 |
| VD87 | BFCT | 4.745842 | 2.791887 | 2.592259 | 1999.101 | 930.6962 | 471.6667 |
| VD89 | BFCT | 4.952374 | 2.154276 | 1.857331 | 770.2941 | 487.6875 | 433.8864 |
| VD92 | BFCT | 1.240298 | 1.775112 | 1.85017 | 1038.795 | 327.8857 | 433.2564 |
| Breastfed infants with vitamin D supplementation, *n* = 15. | | | | | | | |
| VD26 | BFVD | 4.774487 | 1.468577 | 2.347654 | 564.7869 | 811.1475 | 788.5833 |
| VD27 | BFVD | 4.825738 | 1.913252 | 2.345492 | 1802.815 | 769.2174 | 259.1304 |
| VD38 | BFVD | 4.651231 | 2.286242 | 2.12793 | 1688.094 | 646.549 | 482.5532 |
| VD40 | BFVD | 4.860135 | 1.975117 | 1.368477 | 2642.5 | 568.8261 | 441 |
| VD41 | BFVD | 4.814519 | 2.682855 | 2.355611 | 1758.38 | 643.4516 | 464.6667 |
| VD43 | BFVD | 2.405086 | 2.397053 | 2.679175 | 938 | 337.6667 | 431.625 |
| VD46 | BFVD | 4.01004 | 1.521584 | 1.772017 | 532.2162 | 508.5217 | 357.25 |
| VD53 | BFVD | 2.090501 | 1.358324 | 1.567194 | 1755.526 | 569.5283 | 463.7576 |
| VD66 | BFVD | 4.897709 | 1.881613 | 1.718183 | 1129.043 | 332.15 | 466.575 |
| VD71 | BFVD | 4.958285 | 2.712145 | 2.792442 | 2133.722 | 733.5606 | 512.2692 |
| VD73 | BFVD | 1.685837 | 1.834844 | 3.128137 | 1274.857 | 258.6667 | 403.5 |
| VD77 | BFVD | 5.003939 | 2.039138 | 2.336665 | 2228.491 | 419.3333 | 572.9623 |
| VD80 | BFVD | 2.173385 | 3.285815 | 3.44744 | 739.7586 | 548.5526 | 394.8125 |
| VD88 | BFVD | 5.060743 | 2.853259 | 2.586886 | 2699.754 | 490.2826 | 629.6429 |
| VD91 | BFVD | 5.050996 | 1.471596 | 2.570014 | 444.6757 | 430.5278 | 445.0526 |
| Formula-fed infants without vitamin D supplementation, *n* = 11. | | | | | | | |
| VD06 | FFCT | 4.827654 | 2.586501 | 2.472952 | 1170.866 | 828.9828 | 348.6579 |
| VD11 | FFCT | 4.842326 | 1.895106 | 3.167446 | 696.1818 | 489.4103 | 556.7 |
| VD13 | FFCT | 4.799084 | 2.125127 | 2.52725 | 3521.46 | 777.0122 | 772.9589 |
| VD14 | FFCT | 3.117982 | 2.643286 | 1.150146 | 526.4545 | 467.2857 | 448.9231 |
| VD22 | FFCT | 4.682897 | 2.794397 | 1.527285 | 1374.318 | 627.8382 | 549.325 |
| VD33 | FFCT | 4.792996 | 1.692234 | 1.227412 | 621.0189 | 799.0909 | 710.5 |
| VD42 | FFCT | 4.806177 | 2.145223 | 2.7966 | 1295.644 | 574.8491 | 490.1034 |
| VD44 | FFCT | 4.590633 | 1.25375 | 2.653897 | 903.1548 | 568.7083 | 563.2321 |
| VD54 | FFCT | 4.550039 | 1.323156 | 2.508296 | 2089.722 | 550.0612 | 456.6667 |
| VD57 | FFCT | 0.715695 | 1.795263 | 2.52182 | 2770.125 | 654.2553 | 600.1967 |
| VD90 | FFCT | 4.716746 | 1.733439 | 2.81778 | 2840.029 | 685.2286 | 280.7778 |
| Formula-fed infants with vitamin D supplementation, *n* = 20. | | | | | | | |
| VD02 | FFVD | 4.892776 | 2.348236 | 3.315672 | 1599.308 | 1205.5 | 534.1395 |
| VD03 | FFVD | 0.5866961 | 2.698601 | 2.159604 | 607.7258 | 538.4286 | 388 |
| VD07 | FFVD | 3.620889 | 1.828138 | 2.773738 | 1014.385 | 749.3 | 594.0517 |
| VD30 | FFVD | 3.938385 | 1.77295 | 2.175574 | 3235.529 | 1207.5 | 492.6429 |
| VD31 | FFVD | 4.761791 | 2.195864 | 1.644652 | 1165.887 | 793.5 | 704.1154 |
| VD34 | FFVD | 4.721479 | 1.689694 | 3.146079 | 1678.452 | 958.8971 | 938.53 |
| VD47 | FFVD | 4.806448 | 1.433632 | 2.245464 | 1549.28 | 816.4308 | 477.4912 |
| VD61 | FFVD | 4.607327 | 2.617178 | 2.478761 | 1012.813 | 669 | 430.2222 |
| VD62 | FFVD | 1.345758 | 1.310853 | 1.784959 | 1430.923 | 668.2727 | 623.4384 |
| VD63 | FFVD | 4.754459 | 1.076991 | 3.183672 | 730.6522 | 501.3077 | 629.918 |
| VD64 | FFVD | 3.179647 | 1.555402 | 2.046333 | 1096.893 | 432.0909 | 511.12 |
| VD67 | FFVD | 4.730224 | 1.460343 | 2.496368 | 1367.729 | 266.0938 | 474.5682 |
| VD72 | FFVD | 4.649341 | 1.604788 | 1.041251 | 1730.159 | 712.0222 | 529.5714 |
| VD74 | FFVD | 4.692577 | 2.546072 | 3.317625 | 2770.876 | 586.1702 | 485.8864 |
| VD76 | FFVD | 2.942405 | 2.392273 | 2.271278 | 889.4872 | 482.75 | 533.7258 |
| VD82 | FFVD | 4.609276 | 1.430935 | 1.865935 | 2755.045 | 543.5192 | 410.2609 |
| VD85 | FFVD | 4.034173 | 2.490928 | 2.803197 | 1962.531 | 909 | 445.4474 |
| VD86 | FFVD | 4.962796 | 2.495893 | 2.381167 | 1077.279 | 647.4912 | 607.4474 |
| VD93 | FFVD | 4.914546 | 1.582143 | 2.795579 | 1047.04 | 455.5357 | 320.1 |
| VD94 | FFVD | 2.18831 | 1.287892 | 1.831231 | 1025.368 | 512.4688 | 433.9767 |

**Table S4: Taxonomic distribution of bacterial communities in breastfed infants with and without vitamin D supplementation at 4-month-old of age.**

| **Genus** | **BFCT (%)** | | | | | | | | | | | | | | | | **BFVD (%)** | | | | | | | | | | | | | | |
| --- | --- | --- | --- | --- | --- | --- | --- | --- | --- | --- | --- | --- | --- | --- | --- | --- | --- | --- | --- | --- | --- | --- | --- | --- | --- | --- | --- | --- | --- | --- | --- |
|  | **VD55** | **VD65** | **VD52** | **VD49** | **VD20** | **VD89** | **VD87** | **VD48** | **VD28** | **VD92** | **VD32** | **VD16** | **VD29** | **VD36** | **VD08** | **VD83** | **VD46** | **VD88** | **VD41** | **VD71** | **VD91** | **VD66** | **VD27** | **VD40** | **VD38** | **VD73** | **VD43** | **VD53** | **VD80** | **VD26** | **VD77** |
| Bifidobacterium | 87.16 | 65.82 | 64.19 | 57.31 | 57.46 | 52.68 | 38.94 | 37.24 | 29.21 | 40.78 | 34.79 | 33.13 | 32.83 | 30.83 | 25.97 | 18.48 | 78.98 | 73.58 | 62.83 | 58.54 | 57.44 | 55.11 | 53.24 | 51.25 | 50.71 | 49.66 | 48.48 | 45.90 | 41.31 | 27.11 | 19.99 |
| Collinsella | 0.00 | 0.00 | 0.00 | 0.00 | 11.41 | 0.01 | 0.10 | 0.00 | 0.00 | 0.00 | 0.00 | 0.00 | 0.00 | 4.84 | 12.85 | 0.00 | 0.12 | 1.76 | 0.01 | 0.00 | 0.00 | 0.00 | 0.01 | 0.00 | 0.00 | 0.07 | 0.00 | 0.00 | 0.01 | 0.00 | 0.00 |
| Bacteroides | 0.09 | 0.06 | 0.06 | 3.87 | 5.41 | 0.09 | 0.12 | 0.08 | 0.04 | 43.24 | 30.54 | 35.38 | 41.19 | 26.15 | 19.98 | 39.49 | 1.13 | 2.20 | 0.77 | 0.22 | 0.06 | 0.02 | 0.10 | 0.05 | 0.24 | 0.16 | 0.07 | 0.31 | 32.63 | 38.11 | 41.73 |
| Parabacteroides | 0.00 | 0.01 | 0.00 | 0.00 | 0.07 | 0.01 | 0.01 | 0.03 | 0.00 | 0.00 | 4.36 | 0.00 | 0.03 | 9.68 | 6.76 | 0.00 | 0.01 | 0.00 | 0.00 | 0.00 | 0.00 | 0.01 | 0.01 | 0.00 | 0.00 | 0.00 | 0.00 | 0.02 | 0.41 | 0.00 | 4.34 |
| Blautia | 0.00 | 0.00 | 0.00 | 0.00 | 0.01 | 0.00 | 0.00 | 0.01 | 18.71 | 0.00 | 1.72 | 0.31 | 0.00 | 0.00 | 0.00 | 0.00 | 0.00 | 0.06 | 0.00 | 0.00 | 0.00 | 0.01 | 0.01 | 0.00 | 0.00 | 0.00 | 0.00 | 0.04 | 0.00 | 0.00 | 0.00 |
| Enterococcus | 0.71 | 10.78 | 8.96 | 0.58 | 2.43 | 0.29 | 5.35 | 14.40 | 9.79 | 1.68 | 3.68 | 4.43 | 9.89 | 0.70 | 2.23 | 5.47 | 1.29 | 0.18 | 12.16 | 1.40 | 9.99 | 8.89 | 0.02 | 1.19 | 5.35 | 6.14 | 12.34 | 3.54 | 9.59 | 0.49 | 4.57 |
| Lactobacillus | 0.07 | 1.89 | 0.17 | 3.79 | 0.05 | 4.02 | 0.02 | 0.30 | 1.55 | 3.42 | 0.02 | 0.05 | 0.94 | 0.01 | 0.12 | 0.04 | 0.28 | 0.29 | 0.47 | 0.02 | 0.02 | 7.47 | 11.80 | 12.70 | 0.10 | 5.35 | 2.21 | 5.60 | 2.35 | 0.01 | 0.16 |
| Megamonas | 0.00 | 0.00 | 0.02 | 0.07 | 0.00 | 0.00 | 0.04 | 0.00 | 0.00 | 0.00 | 0.00 | 0.00 | 0.00 | 9.11 | 0.00 | 4.35 | 0.02 | 0.63 | 0.00 | 0.03 | 0.01 | 0.01 | 0.03 | 0.02 | 0.00 | 0.08 | 0.02 | 0.00 | 0.00 | 0.00 | 0.01 |
| Megasphaera | 0.00 | 0.00 | 0.01 | 0.00 | 0.00 | 0.00 | 0.00 | 0.00 | 0.00 | 0.00 | 0.00 | 0.00 | 0.00 | 0.00 | 0.00 | 15.95 | 0.00 | 0.00 | 0.00 | 0.07 | 0.02 | 0.00 | 0.00 | 0.00 | 0.00 | 0.00 | 0.02 | 0.00 | 0.00 | 0.00 | 0.00 |
| Streptococcus | 1.58 | 2.41 | 4.22 | 8.27 | 11.13 | 12.75 | 4.14 | 2.93 | 9.59 | 4.92 | 3.20 | 0.30 | 3.14 | 0.16 | 1.53 | 1.30 | 12.74 | 2.78 | 6.68 | 8.13 | 14.38 | 2.31 | 16.44 | 29.80 | 0.24 | 6.38 | 3.72 | 8.55 | 0.95 | 2.34 | 0.50 |
| Vagococcus | 0.01 | 0.02 | 0.05 | 0.00 | 0.01 | 0.20 | 9.44 | 3.04 | 0.05 | 0.05 | 5.12 | 2.56 | 3.29 | 0.01 | 0.28 | 0.03 | 0.00 | 0.03 | 0.00 | 3.87 | 2.00 | 0.00 | 0.00 | 0.00 | 0.01 | 1.72 | 0.00 | 0.03 | 0.62 | 0.01 | 0.07 |
| Veillonella | 0.10 | 0.64 | 0.02 | 0.06 | 3.97 | 2.07 | 6.48 | 3.81 | 3.61 | 0.80 | 7.22 | 13.95 | 0.48 | 0.48 | 1.08 | 0.04 | 0.04 | 0.91 | 1.57 | 2.40 | 1.80 | 13.30 | 16.63 | 0.28 | 0.42 | 17.86 | 9.80 | 19.35 | 0.07 | 0.16 | 0.34 |
| Others | 10.29 | 18.37 | 22.31 | 26.05 | 8.04 | 27.88 | 35.35 | 38.17 | 27.46 | 5.12 | 9.35 | 9.90 | 8.20 | 18.03 | 29.19 | 14.84 | 5.39 | 17.58 | 15.50 | 25.33 | 14.28 | 12.87 | 1.71 | 4.70 | 42.92 | 12.58 | 23.32 | 16.66 | 12.08 | 31.76 | 28.28 |

**Table S5: Taxonomic distribution of bacterial communities in formula-fed infants with and without vitamin D supplementation at 4-month-old of age.**

| **Genus** | **FFCT (%)** | | | | | | | | | | | **FFVD (%)** | | | | | | | | | | | | | | | | | | | | |
| --- | --- | --- | --- | --- | --- | --- | --- | --- | --- | --- | --- | --- | --- | --- | --- | --- | --- | --- | --- | --- | --- | --- | --- | --- | --- | --- | --- | --- | --- | --- | --- | --- |
|  | **VD06** | **VD90** | **VD11** | **VD14** | **VD44** | **VD22** | **VD33** | **VD57** | **VD13** | **VD42** | **VD54** | | **VD67** | **VD82** | **VD72** | **VD30** | **VD03** | **VD07** | **VD61** | **VD64** | **VD86** | **VD76** | **VD74** | **VD34** | **VD94** | **VD31** | **VD02** | **VD85** | **VD93** | **VD47** | **VD62** | **VD63** |
| Bifidobacterium | 81.34 | 73.85 | 62.46 | 41.05 | 34.97 | 50.92 | 33.09 | 29.10 | 27.25 | 31.22 | 14.98 | | 79.60 | 66.25 | 70.37 | 67.15 | 57.08 | 45.09 | 41.22 | 36.94 | 35.83 | 34.80 | 40.10 | 22.99 | 41.61 | 30.69 | 25.86 | 24.50 | 27.44 | 21.11 | 15.46 | 14.46 |
| Collinsella | 0.00 | 0.01 | 0.01 | 0.01 | 3.33 | 0.00 | 4.52 | 0.00 | 1.20 | 0.00 | 0.00 | | 0.00 | 0.00 | 0.00 | 0.74 | 0.00 | 0.84 | 0.00 | 0.00 | 0.00 | 0.00 | 0.00 | 0.02 | 0.00 | 9.38 | 2.42 | 12.60 | 2.64 | 0.00 | 0.00 | 0.01 |
| Bacteroides | 0.07 | 0.05 | 0.10 | 0.15 | 0.07 | 21.94 | 20.43 | 22.92 | 19.52 | 28.40 | 37.66 | | 0.04 | 0.02 | 1.90 | 2.73 | 0.08 | 1.39 | 0.09 | 0.04 | 0.07 | 1.93 | 0.04 | 0.06 | 36.24 | 36.08 | 46.52 | 25.79 | 25.76 | 37.50 | 44.40 | 42.48 |
| Dysgonomonas | 0.00 | 0.00 | 0.00 | 0.00 | 11.79 | 0.00 | 0.00 | 0.00 | 0.01 | 0.00 | 0.00 | | 0.00 | 0.00 | 0.00 | 0.00 | 0.00 | 0.00 | 0.00 | 0.00 | 0.00 | 0.00 | 0.00 | 0.08 | 0.00 | 0.00 | 0.00 | 0.00 | 0.00 | 0.00 | 0.00 | 0.00 |
| Parabacteroides | 0.01 | 0.01 | 0.01 | 0.01 | 0.00 | 0.09 | 4.64 | 0.01 | 8.80 | 0.05 | 0.03 | | 0.00 | 0.01 | 0.00 | 0.20 | 0.00 | 0.41 | 0.00 | 0.00 | 0.00 | 0.21 | 0.00 | 0.00 | 0.00 | 3.02 | 0.00 | 0.00 | 0.00 | 0.00 | 2.55 | 4.38 |
| Prevotella | 0.00 | 0.00 | 0.03 | 0.01 | 0.01 | 0.00 | 0.04 | 0.00 | 5.02 | 0.03 | 0.00 | | 0.00 | 0.01 | 0.03 | 0.06 | 0.00 | 0.08 | 0.02 | 0.00 | 0.02 | 0.49 | 0.01 | 0.02 | 0.00 | 0.01 | 0.27 | 0.00 | 0.00 | 0.00 | 0.01 | 0.00 |
| Blautia | 0.00 | 0.01 | 0.00 | 0.00 | 0.01 | 0.04 | 0.00 | 1.05 | 0.02 | 0.00 | 0.00 | | 0.39 | 0.00 | 0.01 | 0.00 | 0.01 | 0.00 | 0.01 | 0.00 | 0.00 | 0.00 | 0.00 | 0.01 | 0.01 | 0.03 | 0.00 | 0.00 | 0.00 | 0.00 | 10.44 | 0.69 |
| Clostridium | 0.09 | 0.00 | 0.47 | 1.91 | 0.01 | 0.01 | 0.06 | 0.03 | 0.00 | 0.00 | 1.28 | | 0.01 | 0.01 | 0.01 | 0.01 | 0.01 | 1.67 | 0.01 | 7.22 | 4.31 | 2.78 | 0.58 | 1.93 | 0.03 | 0.00 | 0.00 | 0.00 | 0.00 | 0.03 | 0.92 | 0.00 |
| Dorea | 0.00 | 0.00 | 0.01 | 0.00 | 1.24 | 0.00 | 0.00 | 9.42 | 0.00 | 0.01 | 0.01 | | 0.00 | 0.00 | 0.00 | 0.00 | 0.01 | 0.00 | 0.00 | 0.01 | 5.53 | 0.00 | 0.01 | 0.05 | 0.00 | 0.01 | 0.00 | 0.00 | 0.00 | 0.01 | 0.00 | 0.72 |
| Enterococcus | 5.27 | 2.60 | 23.45 | 13.06 | 1.66 | 0.57 | 8.54 | 1.98 | 1.88 | 4.34 | 6.39 | | 0.28 | 8.55 | 5.82 | 1.93 | 18.60 | 9.85 | 5.89 | 12.88 | 5.68 | 4.51 | 5.48 | 2.85 | 2.13 | 3.44 | 6.74 | 4.71 | 0.76 | 6.04 | 2.43 | 0.85 |
| Eubacterium | 0.02 | 0.01 | 0.01 | 0.00 | 2.07 | 0.00 | 1.63 | 10.02 | 4.00 | 0.00 | 0.00 | | 0.00 | 0.00 | 0.00 | 0.00 | 0.00 | 0.28 | 0.00 | 4.11 | 0.00 | 0.01 | 0.00 | 0.02 | 0.00 | 0.00 | 0.00 | 0.00 | 3.55 | 0.00 | 0.00 | 0.00 |
| Faecalibacterium | 0.00 | 0.00 | 0.00 | 0.00 | 0.00 | 0.00 | 0.01 | 0.00 | 0.01 | 0.00 | 0.00 | | 0.00 | 0.00 | 0.00 | 0.00 | 0.00 | 0.00 | 0.00 | 0.00 | 0.00 | 0.00 | 0.00 | 18.44 | 0.00 | 0.00 | 0.00 | 0.00 | 0.00 | 0.00 | 0.00 | 0.00 |
| Klebsiella | 0.00 | 0.01 | 1.77 | 0.13 | 0.01 | 0.09 | 0.11 | 0.05 | 0.02 | 0.02 | 0.09 | | 0.28 | 0.01 | 0.02 | 0.01 | 1.82 | 0.00 | 0.02 | 0.61 | 0.07 | 0.11 | 0.15 | 0.09 | 0.05 | 0.06 | 0.02 | 0.11 | 0.00 | 0.04 | 0.04 | 0.01 |
| Lactobacillus | 1.29 | 0.03 | 0.02 | 0.21 | 0.03 | 1.34 | 0.03 | 0.03 | 0.01 | 1.81 | 0.02 | | 0.39 | 0.01 | 0.01 | 0.02 | 0.03 | 12.40 | 0.09 | 0.03 | 0.02 | 0.02 | 0.02 | 0.01 | 4.96 | 0.02 | 0.01 | 0.15 | 0.01 | 0.02 | 3.07 | 0.01 |
| Megamonas | 0.00 | 0.00 | 0.02 | 0.02 | 0.00 | 0.00 | 0.02 | 1.78 | 0.00 | 0.00 | 0.02 | | 0.00 | 0.00 | 0.00 | 0.00 | 0.00 | 0.00 | 0.01 | 0.00 | 0.00 | 0.00 | 0.00 | 0.00 | 0.00 | 0.00 | 0.01 | 20.80 | 19.19 | 0.00 | 0.00 | 0.02 |
| Megasphaera | 0.00 | 0.01 | 0.02 | 0.00 | 0.00 | 0.00 | 12.82 | 0.00 | 1.91 | 0.00 | 0.00 | | 0.30 | 0.00 | 0.00 | 0.00 | 0.00 | 0.00 | 0.03 | 0.00 | 0.00 | 0.00 | 0.00 | 0.00 | 0.00 | 0.00 | 0.00 | 0.00 | 0.00 | 0.00 | 0.00 | 0.00 |
| Oscillospira | 0.01 | 0.00 | 0.00 | 0.00 | 0.95 | 0.00 | 0.00 | 1.91 | 0.37 | 0.00 | 0.00 | | 0.00 | 0.00 | 0.01 | 0.00 | 0.01 | 0.00 | 0.00 | 0.01 | 4.67 | 1.15 | 1.50 | 2.78 | 0.00 | 0.00 | 0.02 | 0.00 | 0.00 | 0.01 | 0.00 | 0.43 |
| Phascolarctobacterium | 0.01 | 0.00 | 0.00 | 0.01 | 0.00 | 0.00 | 0.02 | 0.00 | 0.00 | 0.00 | 0.03 | | 0.00 | 0.00 | 0.00 | 0.11 | 0.00 | 0.00 | 0.00 | 0.00 | 0.00 | 0.00 | 0.00 | 0.00 | 0.00 | 1.42 | 3.30 | 0.00 | 0.00 | 0.00 | 0.00 | 5.48 |
| Ruminococcus | 0.01 | 0.00 | 0.00 | 0.00 | 2.85 | 0.06 | 0.22 | 0.00 | 8.67 | 0.00 | 0.03 | | 0.00 | 0.00 | 0.00 | 0.09 | 0.01 | 0.02 | 0.00 | 0.00 | 0.00 | 0.30 | 0.10 | 5.97 | 0.00 | 1.36 | 1.57 | 0.00 | 0.00 | 3.62 | 0.00 | 7.61 |
| SMB53 | 0.01 | 0.00 | 0.00 | 0.61 | 0.74 | 0.03 | 0.13 | 0.94 | 0.81 | 0.61 | 4.75 | | 0.00 | 0.00 | 0.00 | 0.00 | 0.00 | 0.01 | 0.00 | 1.88 | 1.25 | 1.32 | 2.30 | 4.16 | 0.00 | 0.01 | 0.22 | 0.24 | 0.00 | 0.00 | 0.00 | 0.99 |
| Staphylococcus | 0.20 | 0.10 | 0.21 | 0.01 | 0.10 | 0.25 | 0.16 | 0.13 | 0.09 | 0.01 | 0.02 | | 2.99 | 0.07 | 0.12 | 0.02 | 0.11 | 0.09 | 0.20 | 0.58 | 0.01 | 0.00 | 0.01 | 0.04 | 0.02 | 0.03 | 0.02 | 0.03 | 0.01 | 0.05 | 0.01 | 0.03 |
| Streptococcus | 1.81 | 17.49 | 3.07 | 0.45 | 1.50 | 6.73 | 3.53 | 9.07 | 5.08 | 5.78 | 10.26 | | 7.12 | 2.48 | 4.63 | 13.26 | 5.93 | 4.73 | 3.94 | 0.28 | 0.41 | 18.89 | 0.55 | 9.14 | 1.05 | 0.69 | 0.66 | 1.71 | 0.09 | 5.46 | 1.98 | 0.29 |
| Vagococcus | 3.85 | 0.02 | 0.01 | 21.24 | 1.99 | 0.65 | 0.03 | 0.98 | 1.93 | 0.60 | 0.00 | | 0.00 | 0.00 | 4.97 | 1.66 | 0.04 | 1.23 | 11.07 | 0.02 | 14.87 | 2.29 | 9.37 | 8.29 | 4.42 | 0.09 | 0.84 | 0.01 | 0.01 | 1.10 | 2.70 | 0.05 |
| Veillonella | 0.33 | 0.02 | 0.05 | 4.92 | 22.80 | 13.42 | 2.09 | 2.72 | 1.72 | 23.32 | 8.44 | | 0.02 | 2.59 | 0.99 | 0.04 | 9.86 | 2.32 | 3.09 | 9.34 | 5.87 | 8.81 | 6.31 | 5.68 | 5.66 | 0.23 | 1.20 | 0.86 | 0.02 | 13.70 | 3.45 | 0.02 |
| Akkermansia | 0.00 | 0.00 | 0.01 | 0.00 | 0.00 | 0.00 | 0.01 | 0.00 | 0.00 | 0.00 | 0.00 | | 0.00 | 0.00 | 0.00 | 0.00 | 0.01 | 0.23 | 0.03 | 0.01 | 0.75 | 0.00 | 22.31 | 0.00 | 0.01 | 0.00 | 0.00 | 0.02 | 18.43 | 0.01 | 0.00 | 0.01 |
| Others | 5.69 | 5.78 | 8.28 | 16.19 | 13.89 | 3.86 | 7.89 | 7.87 | 11.69 | 3.80 | 15.99 | | 8.58 | 19.98 | 11.09 | 11.96 | 6.40 | 19.37 | 34.28 | 26.05 | 20.61 | 22.38 | 11.16 | 17.38 | 3.81 | 13.43 | 10.32 | 8.46 | 2.07 | 11.28 | 12.51 | 21.45 |

**Table S6: Log-transformed ratio of Firmicutes to Bacteroidetes (F/B) ratio among the breastfed and formula-fed infants with and without vitamin D supplementation.**

| **ID** | **Log F/B ratio** | | |  | **ID** | **Log F/B ratio** | | |
| --- | --- | --- | --- | --- | --- | --- | --- | --- |
|  | **0M** | **1M** | **4M** |  |  | **0M** | **1M** | **4M** |
| BFCT | | | |  | FFCT | | | |
| VD08 | 1.055269 | 7.768545 | -0.94927 |  | VD06 | 0.689202 | 5.942243 | 7.105244 |
| VD16 | 1.729703 | 8.889405 | -0.53972 |  | VD11 | 6.570996 | 8.428626 | 7.475283 |
| VD20 | 1.1537 | 6.780466 | 1.737238 |  | VD13 | 1.430789 | 0.42206 | -0.26422 |
| VD28 | 1.007644 | 7.438438 | 9.920244 |  | VD14 | 5.775836 | 8.832602 | 8.05355 |
| VD29 | 1.040629 | 8.429211 | -1.14779 |  | VD22 | 0.805377 | 7.546338 | 0.07755 |
| VD32 | -0.87566 | -1.74367 | -0.51534 |  | VD33 | 4.527304 | 2.393436 | 0.259013 |
| VD36 | 1.255827 | 8.3541 | -1.32694 |  | VD42 | 0.509577 | -0.62045 | 0.396511 |
| VD48 | 0.992823 | 9.015757 | 0.336022 |  | VD44 | 6.82273 | 7.187962 | 1.886314 |
| VD49 | 0.970285 | 5.719876 | 1.910253 |  | VD54 | 1.769715 | 4.121326 | 0.002711 |
| VD52 | 1.321618 | 7.173163 | 7.713568 |  | VD57 | 1.373243 | 9.523027 | 0.899589 |
| VD55 | 1.872681 | -2.82384 | 4.991522 |  | VD90 | 1.453868 | 9.245646 | 8.320752 |
| VD65 | 6.69522 | 9.343497 | 7.09473 |  | FFVD | | | |
| VD83 | 1.35355 | 5.826938 | -0.50044 |  | VD02 | 1.268664 | 0.905046 | -1.57301 |
| VD87 | 1.305369 | 0.1593 | 8.217329 |  | VD03 | 6.036776 | 7.106451 | 8.663819 |
| VD89 | 4.690783 | 9.24207 | 7.593287 |  | VD07 | -4.88984 | -0.28085 | 4.060039 |
| VD92 | 1.426704 | 9.245008 | -1.96474 |  | VD30 | 0.909892 | -0.00066 | 2.565402 |
| BFVD | | | |  | VD31 | 1.307388 | -2.00388 | -1.45024 |
| VD26 | 1.375674 | -3.8755 | -1.91261 |  | VD34 | 1.040028 | 6.982542 | 8.865478 |
| VD27 | 0.989458 | 8.920792 | 8.591981 |  | VD47 | 1.408594 | -2.21296 | -0.39392 |
| VD38 | 1.404075 | 7.431823 | 5.864046 |  | VD61 | 4.496229 | 8.070362 | 7.70535 |
| VD40 | 1.285914 | 8.160884 | 9.714703 |  | VD62 | 1.015426 | -1.28975 | -0.76864 |
| VD41 | 0.176912 | 8.298591 | 4.743645 |  | VD63 | 3.343185 | 4.753094 | -0.77852 |
| VD43 | 0.823853 | 8.501094 | 8.550635 |  | VD64 | 1.648108 | -0.37716 | 9.482916 |
| VD46 | 9.36049 | 7.87296 | 3.949274 |  | VD67 | 2.211271 | 8.746172 | 8.095529 |
| VD53 | 1.140415 | 6.421452 | 6.767909 |  | VD72 | 0.969309 | 6.150627 | 3.112414 |
| VD66 | 1.99661 | 10.4065 | 8.66407 |  | VD74 | 1.210276 | 8.585036 | 9.291288 |
| VD71 | 1.571412 | 9.736226 | 6.336622 |  | VD76 | 1.180416 | -3.30164 | 4.220488 |
| VD73 | 1.384229 | 8.25813 | 7.910559 |  | VD82 | 1.376127 | 5.557297 | 8.58901 |
| VD77 | 1.887818 | -1.83759 | -1.9384 |  | VD85 | 1.092837 | 1.480192 | 0.185835 |
| VD80 | 2.684294 | -1.47557 | -1.18407 |  | VD86 | 6.220278 | 7.965705 | 9.298274 |
| VD88 | 0.940073 | 8.959174 | 1.580132 |  | VD93 | 1.223121 | -0.36969 | 0.027606 |
| VD91 | 1.341803 | 5.782801 | 8.749151 |  | VD94 | 1.706454 | -1.39196 | -0.85456 |

**Table S7: Microbial KO modules enriched in BFVD or BFCT samples.**

| **Modules** | **Enriched Class** | **LDA Score** | **p-value** | **Reporter score** |
| --- | --- | --- | --- | --- |
| ABC transporters | BFVD | 2.207190833 | 0.026856696 | 1.07748481 |
| Nucleotide metabolism | BFVD | 1.209400153 | 0.036170197 | 1.336759043 |
| Ascorbate and aldarate metabolism | BFVD | 1.15627129 | 0.026856696 | 1.030922427 |
| Dioxin degradation | BFVD | 1.141962675 | 0.026856696 | 1.406045884 |
| Retinol metabolism | BFVD | 1.135905231 | 0.010188932 | 1.200499376 |
| Glutamatergic synapse | BFCT | 1.034652368 | 0.039832619 | -1.352575689 |
| Biosynthesis of vancomycin group antibiotics | BFCT | 1.050015156 | 0.036170197 | -1.557435405 |
| Prostate cancer | BFCT | 1.068597284 | 0.008087197 | -4.883555808 |
| Insulin signaling pathway | BFCT | 1.077808253 | 0.019691236 | -1.504743623 |
| Cell division | BFCT | 1.088462286 | 0.036170197 | -1.359498511 |
| Pathways in cancer | BFCT | 1.093494151 | 0.032798814 | -2.266475645 |
| Progesterone-mediated oocyte maturation | BFCT | 1.11183743 | 0.007189517 | -4.475106686 |
| Antigen processing and presentation | BFCT | 1.11183743 | 0.007189517 | -4.475106686 |
| Glycosphingolipid biosynthesis - ganglio series | BFCT | 1.29190059 | 0.014255291 | -2.918866935 |
| Streptomycin biosynthesis | BFCT | 1.300407374 | 0.043804994 | -1.229155247 |
| Polyketide sugar unit biosynthesis | BFCT | 1.338841109 | 0.014255291 | -1.55214834 |
| Glycosphingolipid biosynthesis - globo series | BFCT | 1.360348266 | 0.024251405 | -2.264214856 |
| Glycosaminoglycan degradation | BFCT | 1.405801767 | 0.026856696 | -3.935219352 |
| Methane metabolism | BFCT | 1.406227612 | 0.048106828 | -1.14790695 |
| Histidine metabolism | BFCT | 1.42841258 | 0.032798814 | -1.266442457 |
| Arginine and proline metabolism | BFCT | 1.592412125 | 0.017706066 | -1.219892358 |
| Transcription machinery | BFCT | 1.73774129 | 0.024251405 | -1.379981308 |
| General function prediction only | BFCT | 1.773476383 | 0.048106828 | -1.156527881 |
